# Mutations in *SLC39A14* disrupt manganese homeostasis and cause childhood-onset parkinsonism–dystonia

**DOI:** 10.1038/ncomms11601

**Published:** 2016-05-27

**Authors:** Karin Tuschl, Esther Meyer, Leonardo E. Valdivia, Ningning Zhao, Chris Dadswell, Alaa Abdul-Sada, Christina Y. Hung, Michael A. Simpson, W. K. Chong, Thomas S. Jacques, Randy L. Woltjer, Simon Eaton, Allison Gregory, Lynn Sanford, Eleanna Kara, Henry Houlden, Stephan M. Cuno, Holger Prokisch, Lorella Valletta, Valeria Tiranti, Rasha Younis, Eamonn R. Maher, John Spencer, Ania Straatman-Iwanowska, Paul Gissen, Laila A. M. Selim, Guillem Pintos-Morell, Wifredo Coroleu-Lletget, Shekeeb S. Mohammad, Sangeetha Yoganathan, Russell C. Dale, Maya Thomas, Jason Rihel, Olaf A. Bodamer, Caroline A. Enns, Susan J. Hayflick, Peter T. Clayton, Philippa B. Mills, Manju A. Kurian, Stephen W. Wilson

**Affiliations:** 1 Genetics and Genomic Medicine, UCL Institute of Child Health, University College London, London WC1N 1EH, UK; 2 Department of Cell and Developmental Biology, University College London, London WC1E 6BT, UK; 3 Developmental Neurosciences, UCL Institute of Child Health, University College London, London WC1N 1EH, UK; 4 Department of Cell, Development and Cancer Biology, Oregon Health & Sciences University, Portland, Oregon 97239, USA; 5 Department of Chemistry, School of Life Sciences, University of Sussex, Brighton BN1 9QJ, UK; 6 Division of Genetics and Genomics, Department of Medicine, Boston Children's Hospital and Harvard Medical School, Boston, Massachusetts 02115, USA; 7 Division of Genetics and Molecular Medicine, King's College London School of Medicine, London SE1 9RT, UK; 8 Department of Radiology, Great Ormond Street Hospital for Children NHS Trust, London WC1N 3JH, UK; 9 Developmental Biology and Cancer, UCL Institute of Child Health and Department of Histopathology, Great Ormond Street Hospital for Children NHS Trust, London WC1N 3JH, UK; 10 Department of Pathology, Oregon Health & Science University, Portland, Oregon 97239, USA; 11 Developmental Biology and Cancer Programme, UCL Institute of Child Health, University College London, London WC1N 1EH, UK; 12 Department of Molecular & Medical Genetics, Oregon Health & Science University, Portland, Oregon 97239, USA; 13 Institute of Neurology, University College London, London WC1N 3BG, UK; 14 Alzheimer's Disease Research Centre, Department of Neurology, Harvard Medical School and Massachusetts General Hospital, Charlestown, Massachusetts 02129, USA; 15 Institute of Human Genetics, Technische Universität München, Munich 81675, Germany; 16 Institute of Human Genetics, Helmholtz Zentrum München, German Research Center for Environmental Health, Neuherberg 85764, Germany; 17 Unit of Molecular Neurogenetics, IRCCS, Foundation Neurological Institute ‘C. Besta', Milan 20133, Italy; 18 Department of Medical and Molecular Genetics, University of Birmingham, Birmingham B15 2TT, UK; 19 Centre for Rare Diseases and Personalised Medicine, School of Clinical and Experimental Medicine, College of Medical and Dental Sciences, University of Birmingham, Birmingham B15 2TT, UK; 20 Department of Medical Genetics, School of Clinical Medicine, University of Cambridge, and Cambridge NIHR Biomedical Research Centre, Cambridge CB2 0QQ, UK; 21 MRC Laboratory for Molecular Cell Biology and Cell Biology Unit, University College London, London WC1E 6BT, UK; 22 Department of Metabolic Medicine, Great Ormond Street Hospital for Children NHS Trust, London WC1N 3JH, UK; 23 Department of Paediatric Neurology, Faculty of Medicine, Cairo University Children's Hospital, Cairo 11432, Egypt; 24 Department of Paediatrics, Section of Paediatric Nephrology, Genetics and Metabolism, Unit of Rare Diseases, University Hospital ‘Germans Trias I Pujol', Universitat Autònoma de Barcelona, Badalona 08916, Spain; 25 Department of Paediatrics, Paediatric Neurology and Neonatology Unit, University Hospital ‘Germans Trias I Pujol', Badalona 08916, Spain; 26 Neuroimmunology Group, Institute for Neuroscience and Muscle Research, Kids Research Institute at the Children's Hospital at Westmead, University of Sydney, Westmead NSW 2145, Australia; 27 Department of Neurological Sciences, Christian Medical College Hospital, Vellore 632 004, India; 28 Department of Neurology, Oregon Health & Science University, Portland, Oregon 97239, USA; 29 Department of Pediatrics, Oregon Health & Science University, Portland, Oregon 97239, USA

## Abstract

Although manganese is an essential trace metal, little is known about its transport and homeostatic regulation. Here we have identified a cohort of patients with a novel autosomal recessive manganese transporter defect caused by mutations in *SLC39A14.* Excessive accumulation of manganese in these patients results in rapidly progressive childhood-onset parkinsonism–dystonia with distinctive brain magnetic resonance imaging appearances and neurodegenerative features on post-mortem examination. We show that mutations in *SLC39A14* impair manganese transport *in vitro* and lead to manganese dyshomeostasis and altered locomotor activity in zebrafish with CRISPR-induced *slc39a14* null mutations. Chelation with disodium calcium edetate lowers blood manganese levels in patients and can lead to striking clinical improvement. Our results demonstrate that SLC39A14 functions as a pivotal manganese transporter in vertebrates.

## Abstract (web summary)

Karin Tuschl, Philippa Mills and colleagues report mutations in the manganese (Mn) transporter gene *SLC39A14* in childhood-onset parkinsonism-dystonia. Using functional recapitulation, the authors also show that slc39A14 loss-of-function in zebrafish can lead to Mn dysregulation and locomotor impairment.

## Graphical abstract

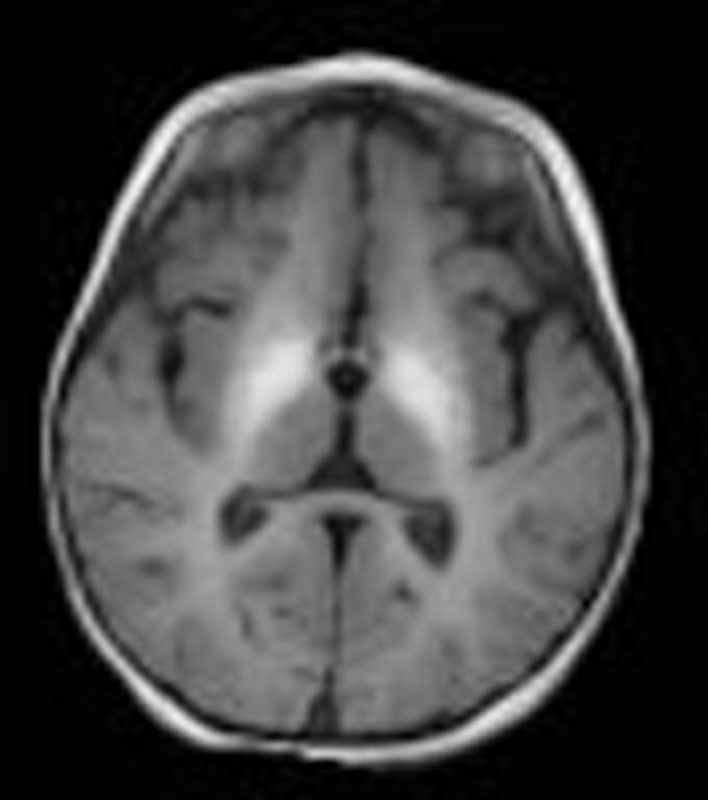

Precise physiological homeostasis of the essential trace metal manganese (Mn) is crucial for normal cell function. Mn acts as a cofactor for multiple enzymes and is involved in numerous physiological processes, including some that are critical for neuronal and glial cell function, such as neurotransmitter synthesis^1^,^2^.

Due to the ubiquity of Mn, nutritional deficiency of this trace metal has rarely been observed^3^. However, mutations in *SLC39A8*, encoding a Mn uptake transporter, have recently been recognized to cause a severe Mn depletion syndrome associated with developmental delay, cerebellar atrophy, epilepsy and short stature^4^,^5^. Biochemically, this disorder is accompanied by dysglycosylation resembling a type II congenital disorder of glycosylation (CDG) due to dysfunction of Mn-dependent enzymes such as the beta 1, 4 glycosyltransferase involved in galactosylation of glycoproteins^4^.

Excessive Mn concentrations on the other hand are neurotoxic and lead to manganism—a parkinsonian movement disorder caused by accumulation of Mn in the basal ganglia^6^. When the homeostatic mechanisms of the gut, which regulates gastrointestinal Mn absorption, and/or the liver, which modulates the biliary excretion of Mn, fail, get bypassed or are overwhelmed, rising Mn blood levels subsequently result in Mn deposition in the brain^6^,^7^. Mn overexposure can occur in welding and mining industries, in individuals using Mn contaminated Ephedrone preparations, and in patients receiving high Mn concentrations in total parenteral nutrition^8^,^9^,^10^. Impaired hepatic Mn excretion in chronic liver disease may cause ‘acquired hepatocerebral degeneration'^11^. Mn toxicity also plays a role in the pathogenesis of other neurodegenerative disorders such as Parkinson's disease (PD)^1^,^12^. Several early-onset parkinsonism genes including *PARK2* (*Parkin*) and *PARK9* (*ATP13A2*) are associated with Mn dyshomeostasis. Affected patients share common neuropathological features of Mn neurotoxicity, including oxidative stress and mitochondrial dysfunction, impaired autophagy and altered alpha-synuclein aggregation^1^,^12^. Indeed, *Parkin* and *ATP13A2* are postulated to protect dopaminergic cells from Mn toxicity^13^,^14^,^15^. Furthermore, polymorphisms in *ATP13A2* are associated with increased susceptibility to Mn toxicity^16^. Alpha-synuclein is also suggested to act as an intracellular Mn store^17^, and Mn exposure promotes alpha-synuclein oligomerization and enhances cellular toxicity^18^,^19^,^20^.

Recently, mutations in *SLC30A10* have been found to cause an inherited disorder of Mn metabolism^21^,^22^. *SLC30A10* encodes a divalent cation transporter expressed on the cell membrane in liver and brain where it mediates efflux of Mn^23^. In affected individuals, Mn accumulates in the basal ganglia and the liver causing parkinsonism–dystonia, chronic liver disease, hypermanganesemia, polycythaemia and abnormal iron indices^21^,^22^. Polymorphisms in *SLC30A10* are associated with increased blood Mn levels and impaired neurological function affecting sway velocity and finger-tapping speed^24^,^25^.

Although the importance of Mn in multiple disease processes is increasingly recognized, transport and homeostatic regulation of Mn *in vivo* are poorly understood. Several transporters have been postulated to play a role in Mn homeostasis and regulation, however, it remains unknown which of these are crucial^4^,^5^,^14^,^23^,^26^,^27^,^28^,^29^,^30^,^31^,^32^,^33^,^34^.

In this study, we have identified a cohort of children with hypermanganesemia and progressive parkinsonism–dystonia who carry homozygous loss-of-function mutations in *SLC39A14*. We show that disodium calcium edetate (Na_2_CaEDTA) effectively chelates Mn and increases urinary Mn excretion in affected individuals. Commenced early in the disease course, chelation therapy can lead to significant improvement of clinical symptoms and, potentially, prevention of disease progression. Our results also demonstrate that SLC39A14 functions as a primary Mn transporter and that mutations in *SLC39A14* impair Mn uptake, without affecting zinc (Zn), iron (Fe) and cadmium (Cd) blood levels. This is further corroborated in zebrafish carrying CRISPR-induced mutations in the *slc39a14* orthologue which also show Mn dyshomeostasis while other metals remain unaffected. Our findings bring new insights into Mn transport and provide a novel zebrafish disease model in which to elucidate the mechanisms underlying Mn-related disease.

## Results

### *SLC39A14* mutations cause Mn-associated neurodegeneration

**Figure 1:**
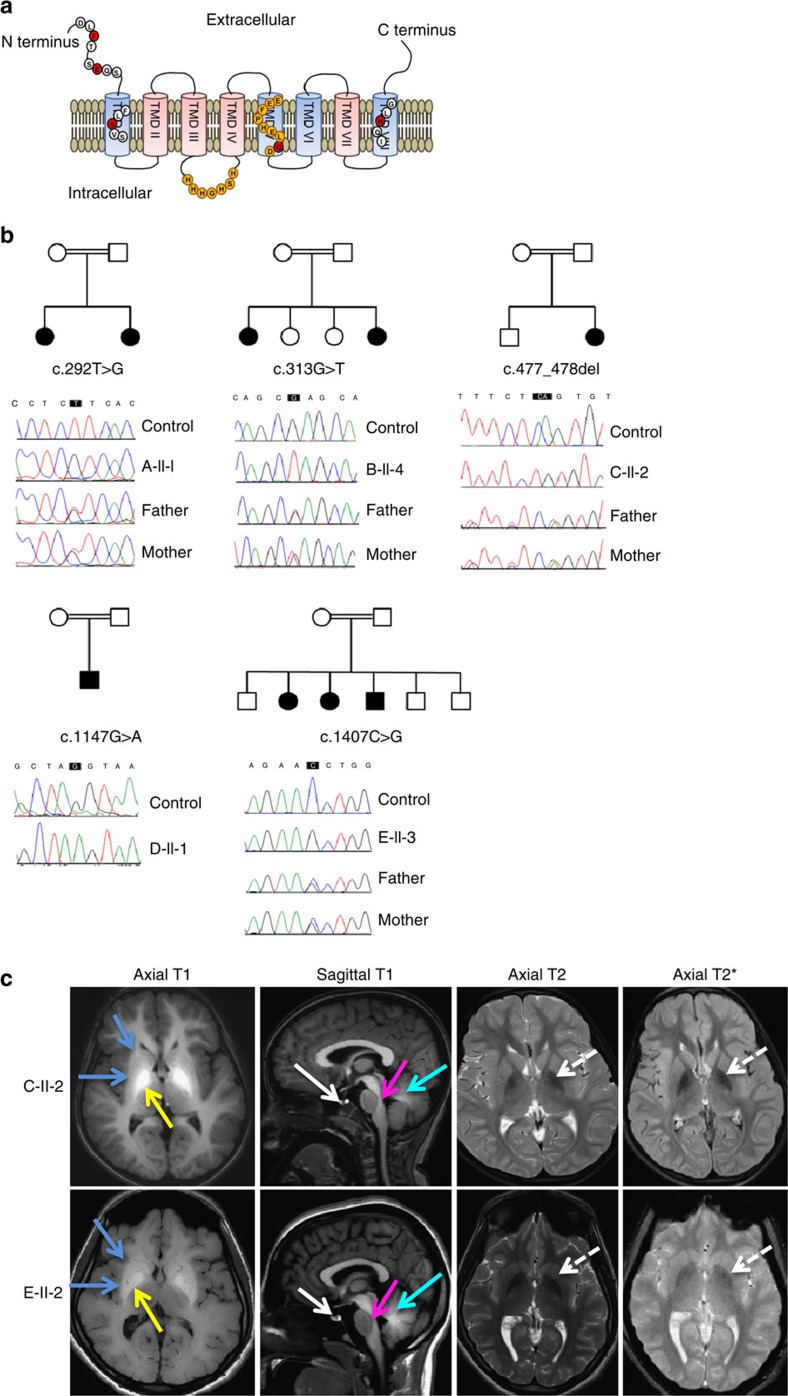
*SLC39A14* mutations lead to cerebral Mn deposition associated with characteristic MRI brain appearances. (**a**) Schematic of SLC39A14 showing its eight TMDs (pink and blue cylinders) interlinked by intracellular and extracellular loops^35^. TMD II, III, IV and VII (pink) are postulated by the transmembrane protein topology prediction tool MemSatSVM (see URLs) to form a pore. The histidine-rich (HXHXHX) and metalloprotease motif (EEXPHEXGD) are highlighted in orange. Mutated amino-acid residues are indicated by red circles. (**b**) Pedigrees and sequence chromatograms of family A-E. Affected individuals are indicated by black shading. Squares represent males, circles females and a double line a consanguineous union. Mutated bases are boxed in black. For each family, the top chromatogram shows the wild-type *SLC39A14* sequence and the hromatogram below the homozygous *SLC39A14* mutation identified in the affected individuals. Parental studies for families A, B, C and E demonstrate that both parents are heterozygous carriers of the identified mutation. (**c**) Representative MRI brain images of patients with *SLC39A14* mutations showing characteristic radiological features: individual C-II-2 aged 3 years and E-II-2 aged 17 years. Generalized T1-hyperintensity of the cerebral white matter, globus pallidus (yellow arrows) and striatum (blue arrows), pituitary gland (white arrows), dorsal pons (pink arrows) and cerebellum (turquoise arrows) can be observed. Hypointensity of the globus pallidus is also evident on T2 and T2*-weighted imaging (white dashed arrows).

SLC39A14 is a divalent metal transporter, also known as Zrt, Irt-like protein 14 (ZIP14), that belongs to the LIV-1 subfamily whose members contain eight transmembrane domains (TMDs), a histidine-rich motif (HXHXHX), and a metalloprotease motif (H/EEXPHEXGD) required for metal transport^35^,^36^ (Fig. 1a). While most SLC39 transporters are specific for Zn there is evidence that SLC39A14 also transports Mn, Fe and Cd^31^,^37^,^38^. Using whole-exome sequencing (WES), in conjunction with autozygosity mapping and Sanger sequencing, we identified homozygous changes in the coding region of *SLC39A14* in eight patients from five unrelated consanguineous families presenting with hypermanganesemia and progressive movement disorder (Table 1, Fig. 1b, Supplementary Fig. 1). The identified mutations include three missense changes (F98V, G383R, N469K) that affect highly conserved amino-acid residues, as well as a nonsense (E105X) and frame-shift mutations (S160Cfs*5) that are expected to generate significantly truncated proteins (Fig. 1a, Supplementary Fig. 2). The G383R mutation is located within the EEXPHEXGD motif required for metal binding^35^. Although it affects the last nucleotide of exon 7 (c.1147G>A), splicing is unaffected (Supplementary Fig. 3). Human *SLC39A14* encodes three isoforms due to alternative splicing of exon 4 and 9 (Supplementary Fig. 4) (ref. 32). The S160Cfs*5 mutation is caused by a 2-bp deletion within exon 4A, solely affecting isoform 2. The clinical phenotype of this individual was not significantly different to that of the remaining patient cohort. Mendelian segregation of the mutations within the family was verified for cases where parent DNA was available (Fig. 1b). None of the identified sequence changes have been reported previously including the dbSNP, 1,000 Genomes Project and ExAC databases (see URLs). Recessive variants in 15 genes causing early-onset PD or Mn dyshomeostasis (Supplementary Table 1) were excluded by analysing the WES data for subject B-II-4, D-II-1 and E-II-4.

Patients with *SLC39A14* mutations showed common clinical features and disease course (Supplementary Note 1, Supplementary Movies 1, 3–5). Affected children presented with loss of developmental milestones, progressive dystonia and bulbar dysfunction in infancy or early childhood. Towards the end of the first decade, they developed severe generalized pharmacoresistant dystonia, spasticity, limb contractures and scoliosis, and lost independent ambulation. Some showed parkinsonian features of hypomimia, tremor and bradykinesia. There appeared to be a degree of cognitive sparing relative to the motor disability. Three children died between 13 months and 7 years, two due to respiratory infections and for the third the cause of death is unknown.

Magnetic resonance imaging (MRI) brain features were characteristic of Mn deposition (Fig. 1c, Supplementary Table 2). Within the deep grey matter, T1-weighted hyperintensity was present in the globus pallidus and, to a lesser extent, the striatum, with thalamic sparing. Generalized T1-hyperintensity indicated extensive white matter involvement including the cerebellum, spinal cord and dorsal pons, with sparing of the ventral pons. Sagittal T1-weighted sequences demonstrated hyperintense signal of the anterior pituitary gland. Axial T2-weighted imaging (T2, T2* and FLAIR sequences) showed hypointensity of the globus pallidus which was progressive in one patient (Supplementary Fig. 5a). Some patients had evidence of cerebral and cerebellar atrophy (Supplementary Fig. 5b,c). Computed tomography imaging undertaken in one patient was normal (Supplementary Fig. 5d).

In all tested patients, whole-blood Mn levels were markedly elevated relative to the reference range (Table 1). Blood levels of Fe, Zn and Cd, divalent metals that can be transported by SLC39A14 in *in vitro* assays^37^,^39^, were assessed in two patients using inductively coupled plasma–mass spectrometry (ICP-MS) and found to be normal when compared with control subjects and literature reference ranges (Table 2) (refs 40, 41). Mn levels were normal in heterozygous parents.

**Figure 2:**
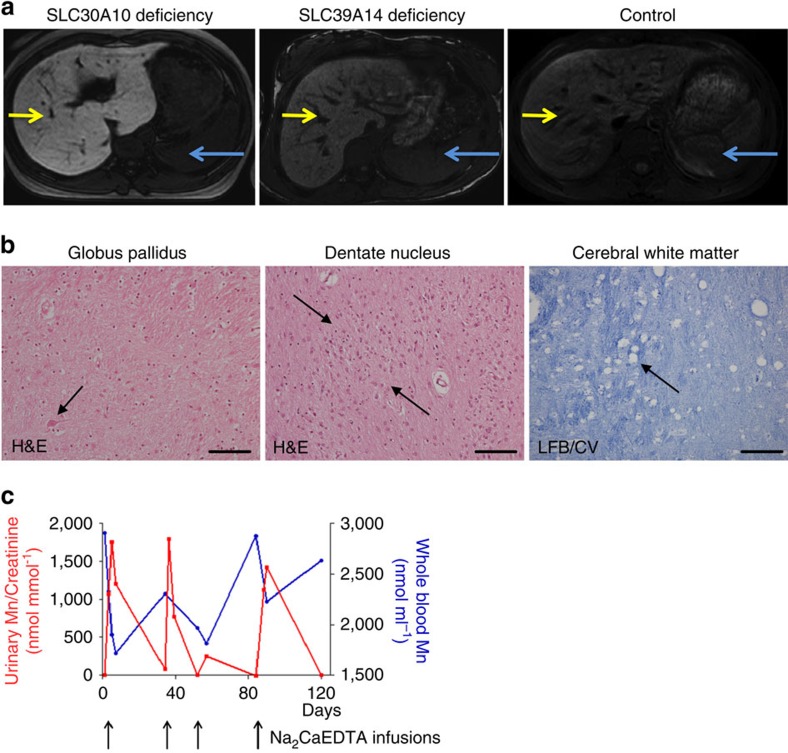
**SLC39A14 deficiency causes hypermanganesemia and neurodegeneration that responds to chelation treatment with Na**_**2**_**CaEDTA.** (**a**) Liver MRIs of a patient with SLC30A10 deficiency, individual E-II-2 with *SLC39A14* mutations and a control subject. The extensive signal hyperintensity on T1-weighted imaging caused by hepatic Mn deposition in SLC30A10 deficiency is absent in individual E-II-2. There is only a subtle degree of T1-hyperintensity when compared with the control subject. Signal intensity of the liver (yellow arrow) was compared with that of the spleen (blue arrow). (**b**) Brain histology from post-mortem examination of subject D-II-1. Sections of globus pallidus and dentate nucleus stained with hematoxylin and eosin (H&E) show marked neuronal loss with only occasional remaining neurons (arrow) accompanied by reactive astrocytosis (shown within the ribbon of the dentate nucleus (between arrows)). Scale bar, 100 μm. Luxol fast blue/cresyl violet stain of a section of the cerebral white matter demonstrates patchy loss of myelin associated with coarse vacuoles (arrow). Scale bar 200 μm. (**c**) Graph showing whole-blood Mn levels and urinary Mn excretion over four courses of Na_2_CaEDTA treatment in individual E-II-2. Arrows indicate timing of Na_2_CaEDTA courses (day 1, 34, 52 and 84). Administration of Na_2_CaEDTA causes a significant increase in urinary Mn excretion (red) accompanied by a drop in whole-blood Mn levels (blue).

In contrast to SLC30A10 deficiency^21^,^22^, children with *SLC39A14* mutations did not develop polycythaemia or liver disease. Liver MRI of individual E-II-4 showed no obvious increase in T1-weighted signal intensity, suggesting that Mn does not accumulate in the liver in this disorder (Fig. 2a). Iron indices including total iron-binding capacity and ferritin were normal. Unlike patients with Mn deficiency due to mutations in *slc39a8* (ref. 4), glycosylation of transferrin, assessed in patient E-II-2, was unaffected despite the Mn overload.

Post-mortem examination performed on individual D-II-1 who died of septic shock following a bronchopneumonia at 4 years of age revealed marked neuronal loss in the globus pallidus (Fig. 2b) while neurons in the caudate, putamen, thalamus and cerebral cortex were relatively well-preserved. Similar severe neuronal loss and gliosis were observed in the dentate nucleus of the cerebellum with relative preservation of the cerebellar cortex. Patchy loss of myelin staining was evident in the cerebral and cerebellar white matter associated with coarse vacuoles (some over 50 μm) and patchy axonal loss on neurofilament staining. A few rounded eosinophilic spheroid structures were noted in the dentate nucleus, but neuropathological features of iron deposition (negative Perls' stain), axonal spheroids, tau and alpha-synuclein pathology were not observed.

### Na_2_CaEDTA lowers blood Mn and alleviates clinical symptoms

In an attempt to reduce the systemic Mn load, individuals C-II-2 and E-II-2 were commenced on chelation therapy with i.v. Na_2_CaEDTA according to a protocol published for SLC30A10 deficiency^42^,^43^. Individual C-II-2, who was started early on in the disease course at 5 years of age, tolerated chelation therapy well without notable side effects and showed dramatic clinical improvement. After 6 months of monthly Na_2_CaEDTA courses (500 mg twice daily for 5 consecutive days) upper limb tremors and athetoid movements had ceased and lower limb dystonia improved to the extent that she regained the ability to walk independently with foot orthoses (Supplementary Movies 1 and 2).

For subject E-II-2, who was commenced on chelation at 17 years of age, Na_2_CaEDTA administration (20 mg kg^−1^ per day) led to marked increase in urinary Mn excretion accompanied by reduction of blood Mn levels (Fig. 2c). Despite mobilization of Mn stores she has continued to deteriorate with worsening tremor and stiffness. Na_2_CaEDTA therefore appears to effectively chelate Mn in this disorder; however, clinical response can be variable, possibly due to patient age and disease severity at treatment onset.

### Tissue expression and Mn transport differ between isoforms

**Figure 3:**
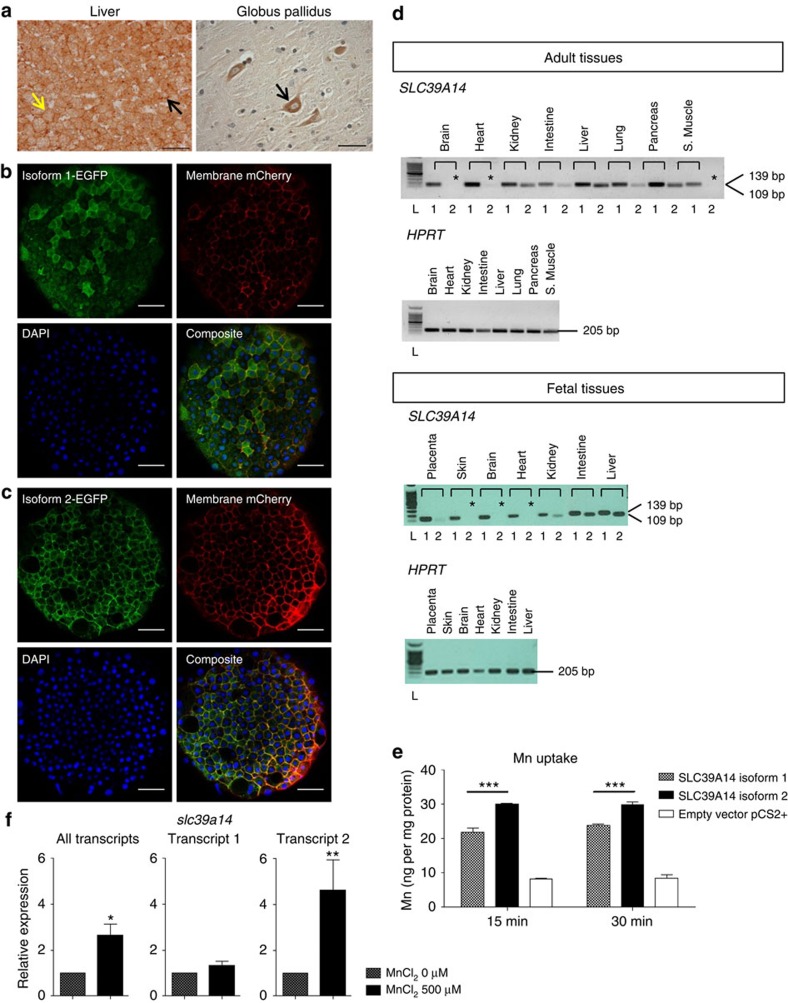
SLC39A14 isoforms 1 and 2 show differences in tissue expression, Mn uptake and transcriptional regulation. (**a**) Immunostaining for SLC39A14 (all isoforms) in healthy control liver shows cell membrane expression (yellow arrow) and punctate cytoplasmic staining (black arrow); scale bar, 50 μm; Abcam anti-SLC39A14 antibody (ab106568, 1:100); and in globus pallidus (GP) from a healthy control shows positively stained large neurons (black arrow); scale bar, 100 μm; Novus anti-SLC39A14 antibody (NBP1-81551, 1:1,000). (**b**,**c**) Confocal images demonstrating the subcellular localization of fluorescently tagged human SLC39A14 isoform 1 (**b**) and isoform 2 (**c**) expressed in zebrafish embryos. Immunostaining for EGFP and mCherry at 6 hpf shows that both isoforms are expressed at the cell membrane (co-localization with membrane mCherry) and in the cytoplasm. 4′,6-diamidino-2-phenylindole (DAPI) was used as a nuclear stain. Scale bar, 50 μm. (**d**) RT–PCR of adult and fetal human tissues showing differences in mRNA expression between isoform 1 (ubiquitous expression in the tissues examined) and isoform 2 (* absent expression in brain, heart, skeletal muscle and skin). Amplicons for isoform 1 and 2 span 139 bp and 109 bp, respectively. Hypoxanthine-guanine phosphoribosyltransferase (HPRT) was used as a housekeeping gene. L, 100 bp ladder (Promega). (**e**) Graph showing Mn uptake in HEK-293 cells transiently transfected with wild-type SLC39A14 isoform 1 and 2, and empty pCS2+ vector following 15 and 30 min of MnCl_2_ (1 μM) exposure. Both isoforms facilitate Mn uptake. Cells transfected with isoform 2 have significantly higher Mn levels (*P*=0.009). Data are presented as means±s.d. from two independent experiments. Statistical analysis was performed using one-way ANOVA (*P*=0.0002 (15 min), *P*=0.0002 (30 min)) and Tukey's multiple comparison test (***P<0.001). (**f**) Graph showing *slc39a14* transcript levels assessed by qRT–PCR in 5 dpf zebrafish larvae after exposure to 500 μM MnCl_2_ for 24 h. Overall transcript levels are increased (*P*=0.035). Transcript levels of isoform 1 are unchanged (*P*=0.41) while those of isoform 2 show a 4.6-fold increase (*P*=0.005). Data are presented as means±s.d. from three independent experiments. Statistical analysis was performed using Student's two-tailed *t*-test on individual ΔCt values (*P<0.05, **P<0.01). ANOVA, analysis of variance.

Immunostaining of sections of human control liver showed that SLC39A14 localized to the cell membrane and the cytoplasm of hepatocytes in a punctate pattern (Fig. 3a). In the brain, SLC39A14 was detected in large neurons, especially in the globus pallidus, insular cortex and dentate nucleus, occasionally in the putamen and rarely in the caudate nucleus (Fig. 3a, Supplementary Fig. 6). Comparable subcellular localization was seen when C-terminally eGFP-tagged human SLC39A14 was expressed in zebrafish embryos. Both isoforms 1 and 2 localize to the cell membrane and the cytoplasm (Fig. 3b,c).

Given that individual C-II-2 carries mutations that only affect isoform 2, tissue expression and function of both SLC39A14 isoform 1 and 2 were examined. The isoforms differ by 20 amino acids encoded by exon 4A and 4B, respectively (Supplementary Figs 4 and 7). Reverse transcription–PCR (RT–PCR) showed that transcript 1 was ubiquitously expressed in all tissues examined while transcript 2 was not detected in the brain (Fig. 3d). Transient expression of wild-type SLC39A14 isoform 1 and 2 in HEK-293 cells confirmed that both facilitate Mn uptake (Fig. 3e). However, isoform 2 showed a greater ability to transport Mn than isoform 1. These results indicate that both isoforms localize to the cell membrane where they facilitate Mn uptake, albeit potentially with different efficacy.

### Mn affects transcriptional regulation of zebrafish *slc39a14*

In other contexts, expression of metal transporters is controlled by the concentration of their substrate thereby ensuring tight regulation of metal levels^44^,^45^,^46^. We assessed the effect of Mn on expression of zebrafish *slc39a14* by quantitative PCR (qPCR). Acute MnCl_2_ exposure (500 μM for 24 h) of zebrafish larvae at 4 days post fertilization (dpf) led to a significant increase in *slc39a14* expression. This increase in expression was caused by upregulation of transcript 2 mRNA levels while levels of transcript 1 remained unchanged (Fig. 3f). Transcriptional regulation of *slc39a14* by its substrate is therefore likely to contribute to Mn homeostasis. Subchronic MnCl_2_ exposure (50 μM, 72 h) on the other hand did not significantly affect *slc39a14* transcript levels (data not shown).

### Missense mutations in human *SLC39A14* impair Mn uptake

**Figure 4:**
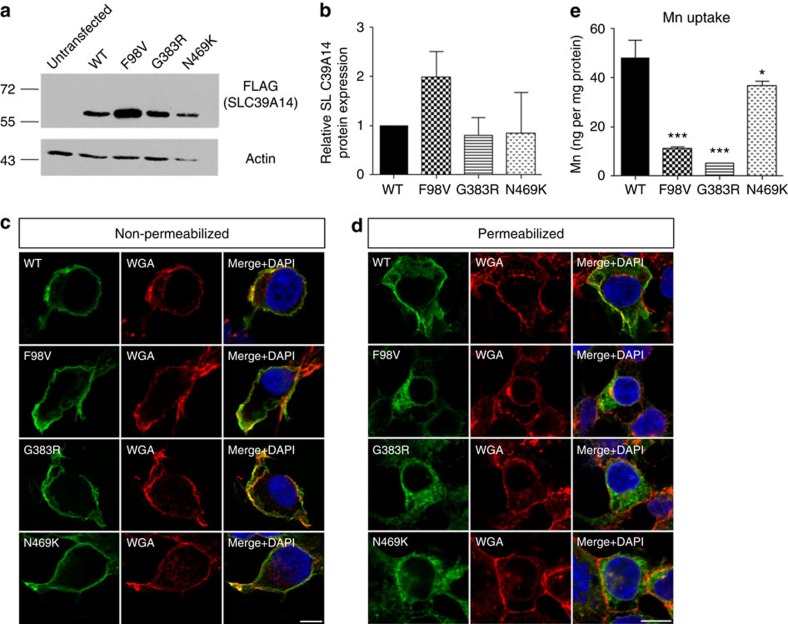
*In vitro* expressed mutant SLC39A14 shows compromised Mn uptake despite normal subcellular localization. (**a**) Immunoblot of whole-cell lysates of stably transfected HEK-293 cells showing expression of the wild-type and three mutant SLC39A14 proteins. Actin was used as a loading control. (**b**) Quantification of SLC39A14 protein levels relative to actin expression revealed no significant difference between wild-type and mutant SLC39A14. Data are presented as means±s.d. from three repeat experiments. Statistical analysis was performed using one-way ANOVA (*P*=0.071). (**c**,**d**) Confocal images of stably transfected HEK-293 cells expressing FLAG-tagged SLC39A14 (green) showing that wild-type and mutant transporters co-localize with wheat germ agglutinin (WGA)-labelled plasma membrane (red) in (**c**) non-permeabilized HEK-293 cells and show additional intracellular localization in (**d**) permeabilized HEK-293 cells. Nuclei are stained with DAPI. Scale bars, 10 μm. (**e**) Mn influx studies in HEK-293 cells stably expressing wild-type (WT) and mutant forms of SLC39A14 show a decrease in Mn uptake for all SLC39A14 mutants (*P*=0.000 [F98V], *P*=0.000 [G383R], *P*=0.02 [N469K]. Data are presented as means±s.d. from three technical replicates. Statistical analysis was performed using one-way ANOVA (*P*=0.000) and Tukey's multiple comparison test (*P<0.05, ***P<0.001). ANOVA, analysis of variance.

The impact of F98V, G383R and N469K variants on SLC39A14 protein localization and function was investigated in stably transfected HEK-293 cells expressing N-terminal FLAG-tagged wild-type and mutant SLC39A14. Immunoblotting studies showed that overall expression of mutant SLC39A14 in total cell lysates was similar to wild-type SLC39A14 (Fig. 4a,b, Supplementary Fig. 8). Immunofluorescence staining of non-permeabilized (Fig. 4c) and permeabilized (Fig. 4d) HEK-293 cells with anti-FLAG antibody confirmed similar subcellular localization patterns for both wild-type and mutant SLC39A14 at the cell surface and within the cytosol. Investigation of mutant SLC39A14 transporter activity following MnCl_2_ exposure (1 μM; 15 min) showed reduced Mn levels for cells expressing mutant protein, particularly mutants F98V and G383R, when compared with those expressing wild-type protein (Fig. 4e).

### *slc39a14* loss-of-function causes Mn disturbance in zebrafish

**Figure 5:**
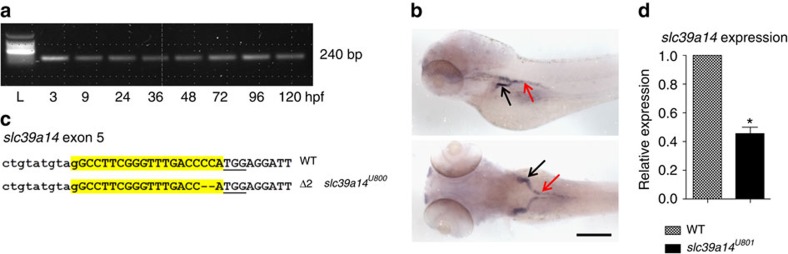
Zebrafish *slc39a14* is expressed during early zebrafish development and significantly reduced in s*lc39a14*^*U801*^ mutants. (**a**) RT–PCR showing *slc39a14* expression between 3 and 120 hpf in zebrafish. L, 100 bp ladder (Promega). (**b**) Whole-mount *in situ* hybridization using a DIG-labelled antisense RNA probe showing *slc39a14* expression in the proximal convoluted (black arrows) and straight (red arrows) pronephric tubules in zebrafish larvae at 4 dpf. Top, lateral view; bottom, dorsal view. Scale bar, 200 μm. (**c**) DNA sequence of the region within exon 5 of *slc39a14* targeted by a CRISPR guide RNA is highlighted in yellow and the 2-bp deletion introduced in the s*lc39a14*^*U801*^ mutant indicated by dashes. Pam sequence underlined. (**d**) qRT–PCR demonstrates a 2.2-fold reduction in *slc39a14* expression in homozygous s*lc39a14*^*U801*^ mutants (*P=0.0117). Primers were designed to detect all *slc39a14* transcripts (Supplementary Table 4). *Ef1α* was used as a reference gene. Data are presented as means±s.d. from three independent experiments. Statistical analysis was performed using Student's two-tailed *t*-test on individual ΔCt values (*P<00.5).

To further elucidate the role of *SLC39A14* in Mn homeostasis, we generated a zebrafish model of Slc39a14 deficiency by CRISPR/Cas9 genome editing. RT–PCR confirmed expression of zebrafish *slc39a14* at all stages of embryonic and early larval development (Fig. 5a). Whole-mount *in situ* hybridization demonstrated *slc39a14* expression in the proximal pronephric ducts at 4 dpf (Fig. 5b, Supplementary Fig. 9). 5′ and 3′ RACE showed that zebrafish, as for human *SLC39A14*, have an alternative fourth and ninth exon and, in addition, an alternative eighth exon encoding a fourth splice isoform (Supplementary Fig. 10). Sequence alignment of human and zebrafish Slc39a14 demonstrated that both proteins share high sequence homology. About 62% of amino-acid residues are identical with a higher degree of conservation observed around the TMDs and the metalloprotease motif (Supplementary Fig. 11), suggesting that the human and fish Slc39a14 transporters are likely to have comparable functions.

A zebrafish mutant line that carries a *slc39a14* null mutation (*slc39a14*^*U801*^) in exon 5, which is shared by all transcripts, was generated using CRISPR/Cas9 genome editing (Supplementary Fig. 12) (refs 47, 48). The mutant harbours a frame-shift mutation P210Hfs*48 (c.629_630del) predicted to truncate the protein by 246 amino acids with loss of the metal-binding domain and TMD III to VIII (Fig. 5c, Supplementary Fig. 11). Because an antibody to zebrafish Slc39a14 is not available, we assessed s*lc39a14* mRNA expression in homozygous *slc39a14*^*U801*^ mutants and found it markedly reduced (Fig. 5d), suggesting nonsense-mediated decay of the mutant transcripts.

**Figure 6:**
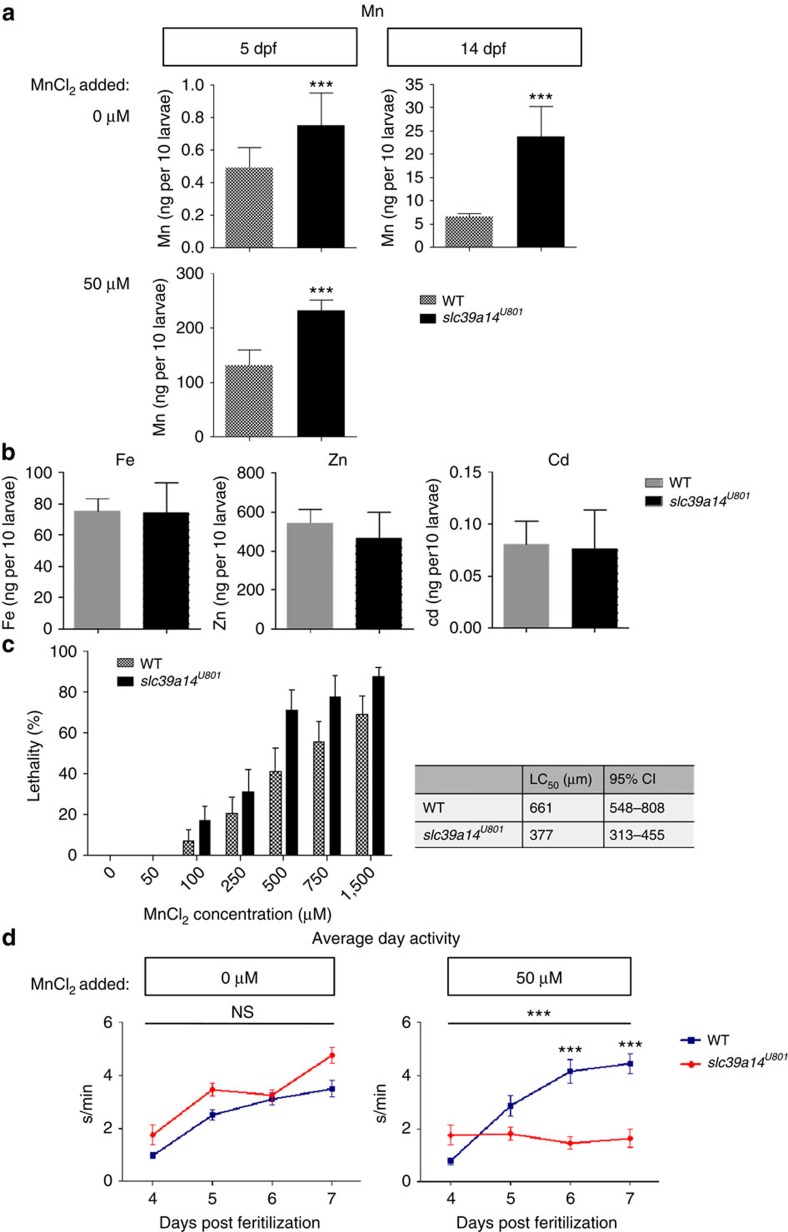
Loss of *slc39a14* function in zebrafish leads to increased Mn accumulation and sensitivity as well as impaired locomotor behaviour. (**a**) Mn levels assessed in homozygous s*lc39a14*^*U801*^ and wild-type (WT) larvae show that mutant larvae have significantly raised Mn levels at 5 dpf (*P*=0.001) and 14 dpf (*P*=0.0002), and Mn accumulation on MnCl_2_ exposure (50 μM from 2 dpf) is significantly higher in mutant compared with WT larvae (*P*=0.000) at 5 dpf. Measurements were taken from pools of 10 larvae. Data are presented as means±s.d. from a minimum of five independent experiments. Statistical analysis was performed using Student's two-tailed *t*-test (***P<0.001). (**b**) Graph showing Fe, Zn and Cd levels in 14 dpf mutant and WT larvae. Levels of all three metals are not significantly different between the two groups (*P*=0.906 [Fe], *P*=0.257 [Zn], *P*=0.834 [Cd]). Measurements were taken from pools of 10 larvae. Data are presented as means±s.d. from five independent experiments. Statistical analysis was performed using Student's two-tailed *t*-test (NS, not significant). (**c**) Graph presenting the lethality in homozygous s*lc39a14*^*U801*^ and WT larvae at 5 dpf on MnCl_2_ exposure between 2 and 5 dpf. Median lethal concentration (LC_50_) of MnCl_2_ determined by Probit regression analysis was 661 μM for WT (95% confidence interval (CI) 548–808 μM) and 377 μM (95% CI 313–455 μM) for mutant fish. Data are presented as means±s.e.m. from nine independent experiments. (**d**) Locomotor behaviour studies of homozygous s*lc39a14*^*U801*^ and WT larvae show that in unexposed conditions there is no significant difference in locomotor activity; and on MnCl_2_ exposure, locomotor activity is markedly reduced in mutant larvae compared with WT. The locomotor behaviour was tracked during 4 and 7 dpf using automated analysis software. s/min, movement in seconds per minute. Data are presented as means±s.e.m. 12 larvae were analysed per condition. Statistical analysis was performed using two way ANOVA (i, *P*=0.18; ii, *P*=0.000) (***P<0.001; NS, not significant). ANOVA, analysis of variance.

Homozygous *slc39a14*^*U801*^ mutants showed a 35% increase in Mn levels at 5 dpf and 72% increase at 14 dpf, confirming a crucial role for Slc39a14 in Mn clearance (Fig. 6a). Other previously reported substrates of the transporter, including Fe, Zn and Cd^37^, were unaffected (Fig. 6b). Larval length did not differ between mutant and wild-type larvae, excluding differences in size accounting for the observed difference in Mn levels (Supplementary Fig. 13). Despite abnormal Mn levels, *slc39a14*^*U801*^ mutants survived into adulthood without any obvious morphological or developmental defects.

Exposure of homozygous *slc39a14*^*U801*^ mutants to a sublethal MnCl_2_ concentration (50  μM) from 2 to 5 dpf led to a much greater accumulation of Mn relative to wild-types at 5 dpf (Fig. 6a). Homozygous *slc39a14*^*U801*^ larvae also showed increased sensitivity to Mn-induced toxicity; the median lethal concentration (LC_50_) of MnCl_2_ was significantly lower for mutant compared with wild-type larvae, and analysis of the relative median potency of MnCl_2_ (1.75, 95% confidence interval 1.2–2.9) suggested MnCl_2_ to be 1.75 times more lethal for mutant than for wild-type larvae (Fig. 6c, Supplementary Fig. 14). A locomotor assay^49^ confirmed that exposure of homozygous *slc39a14*^*U801*^ but not wild-type larvae to MnCl_2_ leads to a prominent reduction in their average locomotor activity (Fig. 6d). These analyses of the *slc39a14*^*U801*^mutant demonstrate that loss of Slc39a14 function causes Mn dyshomeostasis similar to observations in humans.

### Cerebral Mn deposition induces transcriptional changes

**Figure 7:**
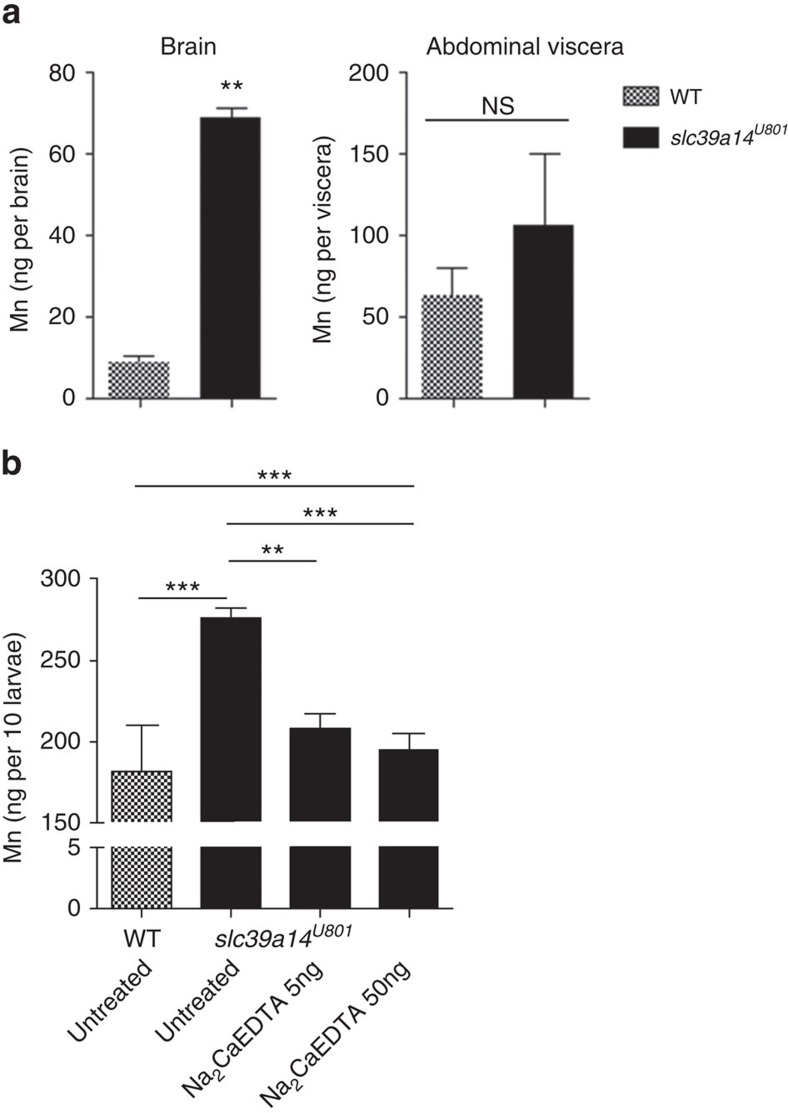
Mn accumulates in the brain of s*lc39a14*^*U801*^ mutants and can be lowered by Na_2_CaEDTA. (**a**) Tissue Mn levels of wild-type (WT) and homozygous s*lc39a14*^*U801*^ zebrafish at 1 year of age show that mutant brain tissue accumulates significantly higher amounts of Mn (*P*=0.0012) while no differences are evident in the Mn content of abdominal viscera (*P*=0.116). Measurements were taken from pools of four brains/abdominal viscera. Data are presented as means±s.d. from two independent experiments. Statistical analysis was performed using Student's two-tailed *t*-test (**P<0.01; NS, not significant). (**b**) Graph showing the effect of Na_2_CaEDTA injections on Mn levels in homozygous s*lc39a14*^*U801*^ larvae. Mutant larvae exposed to 50 μM MnCl_2_ from 2 to 5 dpf were injected into the heart with 5 or 50 ng of Na_2_CaEDTA at 2, 3 and 4 dpf, resulting in a reduction of Mn levels to that of wild-type larvae (*P*=0.000 (WT untreated versus mutant untreated), *P*=0.001 (mutant untreated versus 5 ng), *P*=0.001 (mutant untreated versus 50 ng)). Measurements were taken from pools of 10 larvae. Data are presented as means±s.d. from a minimum of three independent experiments. Statistical analysis was performed using one-way ANOVA (*P*=0.000) and Tukey's multiple comparison test (**P<0.01, ***P<0.001). ANOVA, analysis of variance.

To determine the major site of Mn accumulation in *slc39a14*^*U801*^ mutants, Mn levels were determined in brain and abdominal viscera (intestine, liver, pancreas and spleen) of adult zebrafish at 1 year of age. Mn levels were eight times higher in brains from homozygous *slc39a14*^*U801*^ fish compared with wild-type, whereas no significant differences in the Mn content of abdominal viscera were observed (Fig. 7a). Fe, Zn and Cd levels were unchanged in mutant brain tissue while there was a small increase in Fe in abdominal viscera (Supplementary Fig. 15). Thus, the brain represents the main organ of Mn deposition in *slc39a14*^*U801*^ zebrafish. To assess whether loss of Slc39a14 function and cerebral Mn deposition leads to differential expression of other Mn uptake transporter genes, transcript levels of transferrin receptor 1 (*tfr1a*, *tfr1b*), divalent metal transporter 1 (*DMT1*) and *slc39a8* were compared between mutant and wild-type adult brains and whole larvae (Supplementary Fig. 16). While most of these genes showed no statistically significant changes, transcript levels of *tfr1b* were significantly lower in *slc39a14*^*U801*^ mutant brains. On the other hand, MnCl_2_ overexposure of wild-type and mutant larvae led to upregulation of *tfr1b* but downregulation of *slc39a8* expression. These results suggest that *tfr1b* and *slc39a8* are indeed involved in the regulation of Mn homeostasis, probably with different roles within different tissues.

### Na_2_CaEDTA reduces the Mn load in *slc39a14* ^*U801*^ mutants

To examine whether Na_2_CaEDTA could reduce Mn accumulation in homozygous *slc39a14*^*U801*^ larvae exposed to 50 μM MnCl_2_, we performed daily cardiac injections of Na_2_CaEDTA from 2 to 4 dpf. Na_2_CaEDTA lowered Mn levels similar to those of wild-type fish, confirming the Mn chelating property of Na_2_CaEDTA (Fig. 7b). Addition of the chelator to the fish water did not alter Mn levels (Supplementary Fig. 17) probably due to its poor absorption by the skin and gastrointestinal tract. These data indicate that the *slc39a14*^*U801*^ mutant both recapitulates the human phenotype and offers an effective assay for chelator drug efficacy.

## Discussion

In this study, we have identified a novel autosomal recessive disorder of Mn homeostasis caused by homozygous mutations in *SLC39A14* that lead to early-onset rapidly-progressive parkinsonism–dystonia and neurodegeneration. Identified mutations are predicted to affect transporter function either through nonsense-mediated mRNA decay, protein truncation or impairment of transporter activity. Moreover, we have generated and characterized a zebrafish *slc39a14*^*U801*^ mutant line that recapitulates aspects of the human disease, providing novel insights into Slc39a14 function and facilitating further elucidation of disease mechanisms and drug screening.

SLC39A14 belongs to the SLC39 group of divalent metal transporters. Prior to this study, there was considerable uncertainty with respect to the *in vivo* specificity of this transporter. Unlike most SLC39 proteins that are specific for Zn *in vitro* data suggested that SLC39A14 could transport Mn, Fe and Cd^31^,^37^,^38^. Our data indicate that *in vivo*, the major role for SLC39A14 is to transport Mn. Homozygous *slc39a14*^*U801*^ zebrafish accumulate high levels of Mn and show increased sensitivity to Mn toxicity while levels of Fe, Zn and Cd remain unaffected. This is consistent with our finding that blood metal levels of Fe, Zn and Cd are unaffected in patients with *SLC39A14* mutations. Consequently, SLC39A14 joins SLC30A10 (refs 21, 22, 23) and SLC39A8 (ref. 4, 5) as clinically important, conserved and critical regulators of Mn homeostasis in vertebrates.

Cerebral Mn deposition in patients with *SLC39A14* mutations is associated with characteristic MRI brain appearances similar to those observed in SLC30A10 deficiency. Both disorders present with a progressive extrapyramidal phenotype and neurodegenerative features, including neuronal loss and spongiosis in the globus pallidus, as well as myelin and axonal loss throughout the white matter^50^. However, differences do exist between the two disorders. Individuals with SLC39A14 deficiency do not develop polycythaemia or abnormal iron indices, and liver function is preserved even in cases with advanced neurological disease.

**Figure 8:**
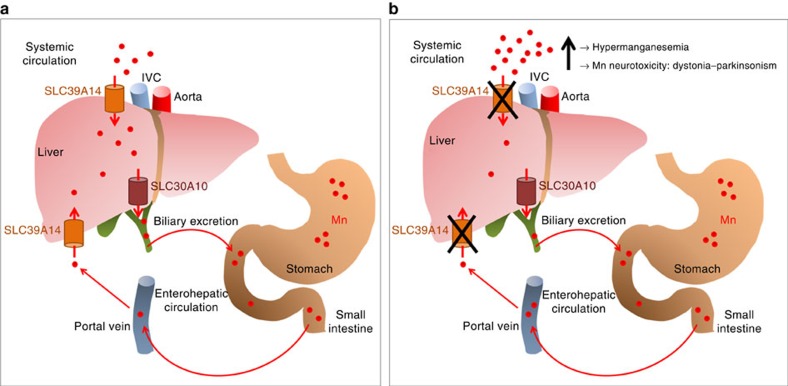
**Proposed disease mechanism in patients with** ***SLC39A14*** **mutations.** (**a**) Under normal conditions nutritional Mn (red) is absorbed in the duodenum and enters the enterohepatic circulation via the portal vein from which it is transported into the liver via SLC39A14, a Mn uptake transporter (orange). Any excess Mn is rapidly removed from the systemic circulation by uptake into the liver via SLC39A14 and excreted into the bile via SLC30A10 (brown), a Mn efflux protein. (**b**) Dysfunction of SLC39A14 (indicated by a black X) impairs hepatic uptake of Mn for subsequent biliary excretion. Consequently, Mn accumulates in the blood and brain leading to hypermanganesemia and neurotoxicity, respectively. IVC, inferior vena cava.

It is plausible that the primary function of SLC39A14 in humans is hepatic Mn uptake for subsequent biliary excretion through SLC30A10. Certainly, MR liver imaging is consistent with lack of hepatic Mn deposition in patients with SLC39A14 deficiency. The high level of SLC39A14 expression in liver tissues (observed in this study and previously^32^,^36^) further substantiates this hypothesis. Mn exposure in mice also causes a marked increase in hepatic *SLC39A14* mRNA expression, further highlighting the role of SLC39A14 in hepatic Mn uptake under a high Mn load^51^. This hypothesis is reinforced by the observation that subject C-II-2 carries mutations that solely affect isoform 2. The absence of brain expression of isoform 2 suggests that the primary defect of Mn clearance does not occur in the brain and that cerebral Mn deposition arises secondarily due to the increased systemic Mn load. As the main regulatory organ involved in Mn homeostasis in humans, the liver seems most likely to play the crucial role in the disease mechanism. *Slc39a14*^−/−^ mice furthermore show transcriptional upregulation of *Slc30a10* expression, suggesting that Slc30a10 and Slc39a14 transporters indeed act in partnership for efficient hepatic Mn detoxification^52^. We postulate that loss-of-function mutations in *SLC39A14* in humans lead to impaired hepatic Mn uptake with resultant hypermanganesemia and downstream neurotoxic effects (Fig. 8).

Whether SLC39A14 is essential for Mn uptake in other tissues remains to be clarified. The ubiquitous expression pattern that we have confirmed for isoform 1 suggests a role for SLC39A14 in a wide range of tissues. Although SLC39A14 is expressed in the intestine, the major site of Mn uptake, intestinal Mn uptake appears to be preserved in this disease as clinically we do not see deficiency of Mn. Similarly, Mn is deposited in the brain, suggesting that transporters other than SLC39A14 facilitate cerebral Mn uptake. Several alternative Mn uptake transporters may compensate for loss of SLC39A14 function. These include DMT1, TfR1, SLC39A8, Mn citrate shuttle, glutamate receptors and various calcium channels/ATPases that have been implicated in Mn transport at the cell membrane either in the brain or the intestine^53^. Quantitative gene expression analysis suggests that *tfr1b*, one of two *tfr1* paralogues in zebrafish and the main cerebral *tfr1* gene^54^, may be involved in facilitating brain Mn uptake in this disorder. Excess Mn in the brain is likely to lead to downregulation of transporter gene expression. On the other hand, increased transcript levels of *tfr1b* on Mn exposure in whole larvae suggests that the role of this gene may vary between tissues.

Our analyses of the subcellular localization of human SLC39A14 in zebrafish embryos and HEK-293 cells demonstrate that SLC39A14 is not only present on the cell membrane but also intracellularly. This is consistent with previous studies in HepG2 cells where SLC39A14 was detected at the plasma membrane and in endosomes^38^. Hence, SLC39A14 might be required for intracellular trafficking of Mn and its transport into specific organelles. This concurs with a recent study showing that SLC39A14 facilitates endosomal trafficking of zinc in enterocytes^55^. Subcellular deficiency of Mn may therefore also contribute to the neuropathology in SLC39A14 deficiency.

Similar to humans, the brain is the main organ of Mn deposition in zebrafish *slc39a14*^*U801*^mutants. Moreover, *slc39a14*^*U801*^ larvae demonstrate impaired locomotor behaviour on Mn exposure. Recently, environmental Mn exposure has been linked to impaired dopamine neuromodulation in zebrafish^56^. The mechanisms that lead to reduced locomotor activity in *slc39a14*^*U801*^ larvae require further studies to elucidate whether, similarly, the observed changes are due to specific Mn neurotoxicity. Unlike humans, however, *slc39a14* is not expressed in the liver during early larval zebrafish development. The prominent expression of *slc39a14* in the pronephric ducts indicates that the kidney is likely to regulate Mn homeostasis in zebrafish larvae.

I.v. Na_2_CaEDTA significantly increases urinary Mn excretion and lowers blood Mn levels in affected individuals. Similarly, in *slc39a14*^*U801*^ zebrafish cardiac injection of Na_2_CaEDTA led to lower Mn levels compared with those of untreated mutants. Nevertheless, clinical response varied between the two individuals given chelation therapy. Subject C-II-2 who was commenced on Na_2_CaEDTA early in the disease course showed a remarkable response with restoration of ambulation, whereas subject E-II-2, severely affected by late stage disease, has continued to clinically deteriorate, even though blood and urine Mn levels suggested mobilization of Mn stores. We know from SLC30A10 deficiency that despite extensive neuronal damage Mn toxicity phenotypes are to a great extent reversible^43^. Although chelation therapy can be efficacious, it could potentially lead to a worsening of neurological symptoms due to mobilization of Mn with a shift into the brain similar to observations in Wilson's disease. Indeed, 10–50% of patients with neurologic Wilson's disease deteriorate during the initial phase of treatment with D-Penicillamine^57^. Furthermore, the type of mutation may have an impact on treatment response. While individual C-II-2 has a homozygous mutation that only affects SLC39A14 isoform 2 all isoforms are affected in subject E-II-2.

We have shown that the tissue localization of isoform 1 and 2 of *SLC39A14* differs and that there may be differences in functional activity. Whilst isoform 1 is ubiquitously expressed with high levels of expression evident in the brain, isoform 2 shows a restricted expression pattern including liver, kidney and intestine – organs involved in the regulation of metal ion homeostasis. As reported previously for murine Slc39a14^32^, our Mn uptake studies in HEK-293 cells confirm that human isoform 2 has a greater ability to transport Mn than isoform 1. Furthermore, Mn-dependent transcriptional regulation of *SLC39A14* expression appears to only affect isoform 2. In zebrafish mRNA expression of transcript 2 is upregulated on Mn loading whereas that of transcript 1 remains unchanged. Similarly, transcription of *SLC30A10* is regulated by Mn and the induction of *SLC30A10* expression on Mn exposure is likely to reduce the body Mn load through promotion of hepatic excretion^22^. These findings suggest that there may be specific roles for the individual SLC39A14 isoforms that might also contribute to differences in treatment response.

Whilst Mn levels have not been assessed in *Slc39a14*^−/−^ mice, alterations in Zn and Fe absorption and trafficking have been reported^55^,^58^. *Slc39A14*^−/−^ mice demonstrate enhanced hepatic iron uptake via transcriptional upregulation of DMT1 and TfR-1 (ref. 58). This is consistent with our finding of increased Fe content in abdominal viscera of *slc39a14*^*U801*^ zebrafish. It is possible that increased DMT1 and TfR-1 expression occurs in response to the high Mn load and indeed *tfr1b* expression increases on Mn exposure in both wild-type and mutant zebrafish larvae. Both of these transporters transport Mn and Fe interdependently^26^,^30^. A recent study of *Slc39a14*^−/−^ mice suggests that Slc39a14 is also instrumental in the uptake of Cd into the liver with hepatic Cd levels being diminished while other organs including kidney, gut and lung showing Cd accumulation^52^.

Our results show that SLC39A14, alongside SLC30A10, is a pivotal Mn transporter required for Mn clearance and maintenance of Mn homeostasis in vertebrates. Our findings expand our understanding of Mn transport and its regulatory mechanisms, and highlight its importance in neurodegenerative disease processes. SLC39A14 is part of a group of Mn transporters that regulates the intricate network of Mn homeostasis. Further studies of the function of this transporter might shed new light on our understanding of how Mn toxicity may contribute to the pathogenesis of PD, with Mn dyshomeostasis not only being observed in inherited disorders of Mn transport and environmental manganism, but also playing an important role in heritable forms of PD that show greater susceptibility to Mn toxicity^6^. However, treatment of Mn neurotoxicity remains unsatisfactory. The *slc39a14*^*U801*^ zebrafish mutant has the potential to illuminate the mechanisms of Mn toxicity and provide new avenues for drug discovery for both rare and common neurodegenerative disorders associated with Mn toxicity.

## Methods

### Subjects

A cohort of patients with extrapyramidal movement disorder, hypermanganesemia and typical MRI brain appearances of Mn deposition (T1-weighted hyperintensity of the globus pallidus and white matter, T2-weighted globus pallidus hypointensity) was analysed in this study. Detailed clinical case descriptions are listed in the Supplementary Information. Written informed consent for DNA storage and genetic analyses was obtained from all subjects/parents. Consent for post-mortem studies of individual D-II-1 was obtained from the parents. The study was approved by the local ethics committees including the West London Research Ethics Committee at the UCL Institute of Child Health, the IRB protocol #7232 at the Oregon Health & Science University, and HREC 10/CHW/114 and 10/CHW/45 at the Kids Research Institute at the Children's Hospital at Westmead.

### Reagents

Chemicals were purchased from Sigma-Aldrich unless stated otherwise.

### WES and linkage analysis

*Family B and D*. Library construction and capture hybridization were performed using either the SureSelect XT Human All Exon V4 or V5 Kit (Agilent Technologies) according to the manufacturer's protocol. Samples were barcoded post-capture to allow for multiplexing of four samples per HiSeq2000 lane. Cluster generation took place on the Illumina cBot according to the manufacturer's recommendations. Sequencing occurred on the Illumina HiSeq2000 using the reagents provided in the Illumina TruSeq PE Cluster Kit v3 and the TruSeq SBS Kit-HS (200 cycle) kit. On average, 96 million pass filter paired-end reads per sample were generated for a mean coverage of 78% at a read depth of 20 ×. Variants were called based on dbSNP139 (ref. 59) and annotation based on SeattleSeq Annotation 137 version 8.01. Filtering was performed using the in-house developed software GEMapp^60^.

*Family A and E*. In the three affected individuals of Family E, a genome-wide linkage scan using the Affymetrix 250 k Sty1 SNP mapping array was carried out on an Affymetrix GeneChip fluidics station 450 according to the manufacturer's instructions. Fluorescence intensities were quantified using an Affymetrix array scanner 3000-7G and the data collected by the Affymetrix GeneChip Operating Software (GCOS) v1.4 (all Affymetrix, Inc.). GTYPE software for BRLMM analysis was used to ascertain genotypes of the samples, which were analysed further using HomozygosityMapper software (see URLs). Significant regions of common homozygosity (>2 Mb) were evaluated by genotyping with microsatellite markers in more detail using an ABI 3,730 DNA Analyzer and Genemapper v3.0 software (Applied Biosystems). The candidate region on chromosome 8 was also genotyped in all available family members of Family A. Primer sequences and the physical order of the markers were obtained from the NCBI database and the UCSC browser (see URLs), respectively. WES was undertaken in affected individual E-II-4. Protein coding exons were captured using SureSelect All Exon 50 Mb Target Enrichment System/SureSelect human All Exon kit (v2; Agilent Technologies). Sequencing was performed on the Illumina GaIIx with 76 bp paired-end reads. Alignment of the obtained sequences to the reference genome (hg19 build) was carried out with Novoalign (Novocraft Technologies Sdn Bhd). Duplicated reads (caused by PCR clonality or optical duplicates) and reads mapping to multiple sites were omitted. Single nucleotide substitutions and small insertion deletions were detected and quality filtered within the SamTools software package and in-house software tools. After filtering out all calls with a read coverage <4 × and a phred-scaled SNP quality of <20, variants were annotated with respect to genes and transcripts with the Annovar tool. Identification of novelty of variants was achieved by comparison to dbSNP132, 1,000 Genomes SNP calls and a subset of 250 control exomes sequenced and analysed by the same method.

### Variant validation and mutation analysis

For sequence validation, mutation screening and segregation analysis, the coding exons and intron/exon boundaries of *SLC39A14* (covering transcripts NM_001128431.2, NM_015359.4 and NM_001135154.1) were PCR amplified from genomic DNA and sequenced bi-directionally using the Big Dye Terminator Cycle Sequencing System (Applied Biosystems) and run on an ABI PRISM 3,730 DNA Analyzer. Primers and PCR conditions are listed in Supplementary Table 3. Population frequencies of identified sequence changes were obtained from the dbSNP, 1,000 Genomes Project and ExAC databases (access date January 2016).

### ICP-MS analysis of metal ions

About 50 μl of EDTA blood were added to 1.95 ml of 3% nitric acid (Fisher) and incubated at 85 °C for 4 h. After centrifugation, the supernatant was analysed by ICP-MS. To determine metal levels in zebrafish larvae, 10 larvae of the same genotype, terminally anaesthetized with MS-222, were pooled and washed several times with dH_2_O. Samples were digested in 1 ml 3% nitric acid at 85 °C overnight followed by a final 95 °C incubation step for 2 h. Tissues (brain and abdominal viscera including intestine, liver, pancreas and spleen) dissected from adult zebrafish were digested in concentrated nitric acid at room temperature overnight and subsequent incubation at 95 °C for 30 min. Samples were diluted with ICP-MS grade H_2_O (Fluka) to a final nitric acid concentration of 3%. Samples from four zebrafish of the same genotype were combined to ensure metal concentrations exceeded the limit of detection. Transiently transfected HEK-293 cells (transfection was carried out 24 h prior analysis) or stable cell lines were incubated in culture medium containing 1  μM MnCl_2_ for 15 min and 30 min, respectively. Following two washes with ice-cold phosphate buffered saline (PBS), cells were harvested in 500 μl PBS using a cell scraper and transferred to a microcentrifuge tube. The cell pellets were washed once in 250 μl ICP-MS grade H_2_O and lysed by repeated freeze-thawing using an ethanol dry ice and 37 °C water bath. The protein concentration was determined using BCA reagent (Thermo Scientific) and the cell lysates digested in nitric acid at a final concentration of 3% in a final volume of 1 ml. Digestion was carried out at 85 °C overnight and 95 °C for 2 h the following day. The metal ion isotopes Mn-55, Fe-56, Zn-66 and Cd-111 were measured by an Agilent 7,500 Series ICP-MS in Helium collision mode. Calibration solutions were prepared for each element between 0 and 200 ng ml^−1^.

### Histopathology and SLC39A14 immunohistochemistry

Paraffin-embedded brain tissue sections were analysed using routine diagnostic histological stains. SLC39A14 immunohistochemistry was performed on control liver sections using a rabbit polyclonal antibody to SLC39A14 (1:100, ab106568, Abcam) on a Leica Bond Max automated system using commercial reagents. Bond Epitope Retrieval Solution 2 (pH∼9 (Leica) was used for antigen retrieval and 3,3′-diaminobenzidine (DAB)/horseradish peroxidase (HRP) for visualization. Immunohistochemistry on normal human brain tissue from the Oregon Brain Bank was performed by hand using the rabbit polyclonal anti-SLC39A14 antibody (1:1,000; NBP1-81551, Novus) with DAB/HRP development as above. This antibody was also used to confirm expression in control liver tissue that was observed using the Abcam antibody (not shown).

### RNA extraction and qRT–PCR

Total RNA was extracted from tissues or pools of 30 zebrafish embryos/larvae using the TRIzol protocol (Life Technologies). Total RNA from human small intestine and foetal liver was purchased from Clontech and Stratagene, respectively. cDNA was synthesized with SuperScript III First Strand Reverse Transcriptase (Life Technologies) using 1 μg total RNA per reaction. In addition, a human cDNA panel from heart, brain, lung, liver, kidney and pancreas was purchased from Clontech. Foetal cDNA from placenta, skin, brain, heart, kidney, intestine and liver were kindly provided by the BabyBioBank at UCL Institute of Child Health, London, UK. Primers and thermocycling conditions used for RT–PCR are listed in Supplementary Table 4.

Real-time PCR was carried out on a CFX96 Touch Real-Time PCR Detection System (Bio-Rad) with the following thermocycling conditions: 2-min initial denaturation at 94 °C followed by 40 cycles of 15 s at 94 °C, 30 s at 60 °C, 30 s at 72 °C. Reactions (final volume of 20 μl) contained 10 μl GoTaq qPCR Master Mix (Promega), 2 μl cDNA and 500 nM of each primer. All reactions were performed in triplicates. Primer efficiency (10^−1/gradient^) was calculated by standard curve analysis using 1:5 serial dilutions over five samples. Primer sequences and efficiencies are listed in Supplementary Table 5. Relative quantification of gene expression was determined using the 2^−ΔCt^ method^61^, with elongation factor 1α (*ef1α*) as a reference gene.

### Cloning of human *SLC39A14*

(i) For transient transfection of HEK-293 cells and expression studies in zebrafish embryos: the coding sequences of human *SLC39A14* transcript 1 and 2 were amplified from foetal liver cDNA with Q5 High Fidelity DNA polymerase (NEB) according to the manufacturer's recommendations using phosphorylated primers (Fwd 5′-CTGGGACCTTGCGGTGAG-3′ and Rev 5′-CTTCCAGTCCCACAGGCTCT-3′) and cloned into pCS2+ vector linearized with StuI (NEB) and dephosphorylated with thermosensitive alkaline phosphatase (Promega). *Escherichia coli* XL10 Gold Ultracompetent Cells (Agilent Technologies) were transformed with amplification products and the success of mutagenesis confirmed by DNA sequencing. To generate C-terminally EGFP-tagged constructs of *SLC39A14* the In-Fusion HD Cloning Kit (Clontech) was used. In brief, the above pCS2+ constructs containing the open reading frame of *SLC39A14* transcript 1 and 2 were amplified by PCR. The EGFP sequence together with a flexible linker (5′-GGTGGATCAGGAGGTGGCGGAAGTGGTGGAGGGAGCTCAGGA-3′) was PCR-amplified from a construct available from previous studies using primers with 15 bp overhangs homologous to the pCS2+ constructs (primer sequences in Supplementary Table 6). In-Fusion cloning reactions and transformation of Stellar Competent *E. coli* cells (Clontech) were performed according to the manufacturer's instructions and construct sequences verified by Sanger sequencing.

(ii) For stable transfection of HEK-293 cells: the pCMV-Entry Vector encoding hSLC39A14 (isoform 1) with myc-FLAG sequence at the C-terminal region was purchased from Origene. Site-directed mutagenesis was performed to generate hSLC39A14 mutants by using the QuikChange Lightning Kit (Stratagene) (primers in Supplementary Table 7). Mutagenesis products were verified by DNA sequencing. Thus generated plasmids containing each of the three missense mutations (F98V, G383R and N469K) were utilized for stable transfection of HEK-293 cells.

### Overexpression of *SLC39A14* in zebrafish embryos

Following linearization of SLC39A14-EGFP constructs with SnaBI (NEB), capped mRNA was generated using the mMessage mMachine Sp6 Transcription Kit (Life Technologies) and purified using the RNeasy Mini Kit (Qiagen). About 50 pg of either *SLC39A14* mRNA (transcript 1/2) was co-injected with 50 pg mRNA encoding membrane mCherry. Injected embryos were fixed at 6 hpf in 4% paraformaldehyde at 4 °C overnight. Following several washes in PBS/0.1% Triton X-100 embryos were blocked in 10% goat serum at room temperature for 1 h. This was followed by incubation with primary antibody (chicken anti-GFP (1:500, ab13970, Abcam), rabbit anti-RFP (1:1,000; PM005, MBL)) at 4 °C overnight. Following washes in PBS/0.1% Triton X-100, embryos were incubated in fluorescent secondary antibody (goat anti-chicken Alexa Fluor 488 (1:200, A-11039, Life Technologies), goat anti-rabbit Alexa Fluor 568 (1:200, A-11011, Life Technologies)) at 4 °C overnight. DAPI (1:500, Life Technologies) was used for nuclear staining. Embryos were mounted in 1.5% low melt agarose and imaged on a Leica TCS SPE confocal microscope using a × 25 0.95 water-immersion objective. Z-stacks were acquired in 1-μm intervals and maximum-intensity projections generated with Fiji software^62^.

### Transient/stable transfection of HEK-293 cells

HEK-293 cells were grown in Dulbecco's Modified Eagle's Medium (DMEM) supplemented with 4.5 g l^−1^ glucose, 2 mM L-glutamine, 10% FBS (PAA Laboratories) and MEM non-essential amino-acid solution (Sigma) and maintained in an incubator at 37 °C and 5% CO_2_. For transient transfection, cells were transfected with 8 μg of plasmid DNA (pCS2+ vector alone or pCS2+ vector containing *SLC39A14* transcript 1 or 2) using Lipofectamine 2,000 (Life Technologies) according to the manufacturer's instructions 24 h after seeding in six-well plates. To generate stable cell lines, HEK-293 cells were transfected with 2 μg plasmid DNA (myc-FLAG-wild-type/292T>G/1147G>A/1407C>G *SLC39A14* transcript 1 in pCMV-Sport6 vector) on day 1 using Effectene reagent (Qiagen), and on day 3 cells were selected with DMEM containing 800 μg ml^−1^ Geneticin (G418). Selected clones were screened by immunoblotting analysis with M2 anti-FLAG-HRP antibody (A8592, Sigma). Positive cell clones were maintained in DMEM supplemented with 10% FBS and 800 μg ml^−1^ G418. One clone per genotype was selected for downstream experiments.

### Immunofluorescence of HEK-293 cells

For confocal studies, stably transfected HEK-293 cells were incubated with Alexa Fluor 647 conjugate of wheat germ agglutinin (1:200 Invitrogen) for plasma membrane labelling for 30 min on ice. Cells were fixed with 4% paraformaldehyde (TAAB Laboratories Equipment Ltd.) in PBS for 10 min followed by washing with PBS and then quenching of free aldehyde groups with ammonium chloride (50 mM) for 10 min. After blocking with 10% foetal calf serum (FCS) in PBS for cell surface staining and 10% FCS and 0.1% Triton X-100 in PBS for permeabilized conditions for 30 min, myc-FLAG-tagged protein was immunostained with the mouse monoclonal antibody anti-Flag M2 (1:500, F3165, Sigma) in PBS with 10% FCS (cell surface staining) and PBS with 10% FCS and 0.1% Triton X-100 (permeabilized conditions) at 4 °C overnight, respectively. Subsequently, cells were washed with PBS and treated with goat anti-mouse Alexa Fluor 488 secondary antibody (1:500, A-11001, Life Technologies) in PBS with 10% FCS (cell surface staining) and PBS with 10% FCS and 0.1% Triton X-100 (permeabilized conditions) for 1 h at room temperature followed by nuclear staining with DAPI (1:5,000; Life Technologies). Coverslips were mounted with ProLong Gold antifade reagent (Life Technologies) on glass slides. Microscopic images were captured using a Leica TCS SP5 AOBS confocal microscope with a × 63/numerical aperture 1.3 oil immersion objective and × 3 optical zoom; the pinhole was set to 1 Airy unit. A series of optical sections were gathered and analysed with the Fiji software^62^.

### Immunoblot analysis

Stably transfected HEK-293 cells were washed twice with cold PBS and lysed in NETT buffer (150 mM NaCl, 5 mM EDTA, 10 mM Tris,1% Triton X-100 and 1 × Complete Mini protease inhibitor Mixture (Roche), pH 7.4). Protein concentrations of the cell lysates were measured using the RC DC Protein Assay kit (Bio-Rad). Cell lysates were mixed with 1 × Laemmli buffer and incubated for 30 min at 37 °C. Proteins were separated electrophoretically on an SDS/10% polyacrylamide gel, transferred to nitrocellulose. Blots were incubated for 1 h in blocking buffer (5% non-fat dry milk in Tris-buffered saline-Tween 20) and then probed for 1 h with mouse anti-FLAG-HRP, M2 (1:10,000; A8592) in blocking buffer. To confirm equivalent loading, blots were stripped for 15 min in Restore PLUS Western Blot Stripping Buffer (Thermo Scientific), blocked for 1 h and reprobed with mouse anti-actin (1:10,000; MAB1501, Millipore) and then goat anti-mouse secondary antibody conjugated to HRP (1:5,000; 12–349, Millipore). Quantification of SLC39A14 protein levels was carried out using ImageJ software. To compare WT and mutant protein levels, intensity values of WT and mutant bands of SLC39A14 and of actin as reference protein were determined. The ratio between WT and mutant values of the reference protein was used as a normalization factor. The mutant SLC39A14 values were then corrected by multiplying them with the corresponding normalization factor. The expression of the mutant protein was represented relative to the WT protein by dividing the corrected mutant intensity values by WT intensity value.

### Zebrafish strains and husbandry

Zebrafish (*Danio rerio*) were raised under standard conditions at 28 °C and staged according to the study by Kimmel *et al*^63^. *AB* and *Tübingen* wild-type strains were used in this study. For whole-mount *in situ* hybridization, zebrafish were treated with 0.002% phenylthiourea from 24 hpf to inhibit pigment formation. Ethical approval for zebrafish experiments was obtained from the Home Office UK under the Animal Scientific Procedures Act 1986.

### Zebrafish whole-mount *in situ* hybridization

Zebrafish larvae were fixed in 4% paraformaldehyde in PBS at 4 °C overnight. A template for *in vitro* transcription was generated by PCR using a reverse primer that contains a T7 promoter sequence (Supplementary Table 8). A digoxygenin (DIG)-labelled antisense RNA probe of 940 bp was synthesized using the DIG labelling kit (Roche) and T7 RNA polymerase (Promega) according to the manufacturer's recommendations. The probe was detected with anti-DIG-AP antibody (1:2,000; 11093274910, Roche) and nitroblue tetrazolium chloride (NBT)/5-bromo-4-chloro-3′-indolyphosphate (BCIP) substrate (Roche) according to published protocols^64^.

### 5′ and 3′ RACE of zebrafish *slc39a14*

Total RNA was extracted from zebrafish larvae at 3 dpf and cDNA ends amplified using the FirstChoice RLM-RACE Kit (Life Technologies) according to the manufacturer's recommendations. Gene-specific outer and inner primers are given in Supplementary Table 9. For subsequent cloning, the inner primers were phosphorylated with T4 polynucleotide kinase (Promega) and the PCR amplicons ligated into pBSK-vector linearized and dephosphorylated with EcoRV (NEB) and thermosensitive alkaline phosphatase. Plasmids were sequenced using M13 forward and reverse primers.

### Generation of zebrafish *slc39a14* null mutant

A blast search (https://blast.ncbi.nlm.nih.gov/Blast/) to the zebrafish genome suggested that only one *slc39a14* orthologue (NC_007116.6) exists in zebrafish. The CRISPR design tool (see URLs) was used to identify a target region in exon 5 of zebrafish *slc39a14* that is present in all transcripts (Fig. 4d)^65^. Oligonucleotides with compatible overhangs (Fwd 5′-TAGGCCTTCGGGTTTGACCCCA-3′ and Rev 5′-AAACTGGGGTCAAACCCGAAGG-3′) were annealed as described by Hwang *et al*.^47^ and cloned into the qDR274 vector (Addgene) digested with BsaI (NEB). The construct was sequence-verified and gRNA generated using the HiScribe T7 High Yield RNA Synthesis Kit (NEB) followed by DNase I (NEB) digestion and purification with RNeasy MiniKit (Qiagen). The Cas9 encoding plasmid pT3TS-nCas9n (Addgene)^48^ was linearized with XbaI (NEB) and capped mRNA generated with the mMessage mMachine T3 Transcription Kit (Life Technologies) followed by polyadenylation with the Poly(A) Tailing Kit (Life Technologies). The synthesized mRNA was purified using the RNeasy MiniKit. Cas9 mRNA and gRNA were co-injected into one-cell stage embryos at a concentration of 200 ng and 75 ng per embryo, respectively.

### High-resolution melting analysis

Genomic DNA was extracted from single embryos at 1 dpf or fin clips from adult zebrafish by incubation in 25 or 50 μl base solution (1.25 M KOH and 10 mM EDTA) at 95 °C for 30 min followed by addition of 25 or 50 μl neutralization solution (2 M Tris HCL). High-resolution melting analysis^66^ was performed to assess the rate of mutagenesis in F0 and F1 embryos and to identify adult F1 heterozygous carriers using Precision Melt Supermix (Bio-Rad) on a CFX96 Touch Real-Time PCR Detection System (Bio-Rad) according to the manufacturer's recommendations. Primers used were Fwd 5′-CCCTGTATGTAGGCCTTCGG-3′ and Rev 5′-CCAAACACGACTGCGGACTTGG-3′. Data were analysed with Bio-Rad Precision Melt Analysis software and the melt curve from injected zebrafish compared with uninjected wild type (Supplementary Fig. 12a). To assess the type of mutations introduced, a region of 330 bp around the CRISPR target site was PCR-amplified with Taq DNA polymerase (Invitrogen) using primers Fwd 5′-AACCCCAAACATCTGAACAGT-3′ and Rev 5′-ACCGGAACAGACCATCAGTT-3′, cloned into pCRII-TOPO vector (Life Technologies) and sequenced (Supplementary Fig. 12b).

### KASP genotyping

For rapid genotyping of mutant zebrafish harbouring the c.629_630 deletion, a mutant allele-specific forward primer 5′-GGGCACATAATAATCCTCCATGGT-3′, a wild-type allele-specific forward primer 5′-GGCACATAATAATCCTCCATGGG-3′ and a common reverse primer 5′-CCCTGTATGTAGGCCTTCGGGTT-3′ were used (LGC Genomics). PCR amplification was performed using KASP Master mix (LGC Genomics) according to the manufacturer's instructions. Fluorescence was read on a CFX96 Touch Real-Time PCR Detection System (Bio-Rad) and the allelic discrimination plot generated using Bio-Rad CFX Manager Software.

### LC_50_ determination of MnCl_2_ in zebrafish larvae

Exposure of wild-type and s*lc39a14*^*U801*^ embryos was commenced at 48 hpf and continued for 3 days. Treatments with MnCl_2_ were performed in 6-well culture plates with 20 embryos per well. MnCl_2_ was added directly to the fish water at the concentrations 50, 100, 250, 500, 750 and 1,500 μM. Dead larvae were counted at 5 dpf. LC_50_ concentrations and relative median potency for wild-type and mutant zebrafish larvae were determined by Probit regression analysis using IBM SPSS Statistics package version 21.

### Locomotor zebrafish behaviour assay

The behavioural assay was conducted as described previously^49^. In brief, zebrafish embryos and larvae were raised on a 14:10-h light/dark cycle. Single larvae were transferred to each well of a flat-bottom, clear polystyrene 96-well plate (Whatman) in fish water (650 μl) at 4 dpf. The 96-well plate was maintained at a constant temperature (28.5 °C) and exposed to a 14:10-h white light/dark schedule with constant infrared illumination within a custom-modified Zebrabox (Viewpoint Life Sciences). The locomotor behaviour of zebrafish larvae was tracked from 4 to 7 dpf using an automated video tracking system (Viewpoint Life Sciences). Larval movement was recorded using Videotrack Quantization mode. The Videotrack detection parameters were empirically defined for clean detection of larval movement with minimal noise. A custom-designed Matlab code was used to extract the average activity data of each larva. Mn exposure was achieved by adding MnCl_2_ (50 μM) directly to the fish water. Results from genotypically presorted larvae were confirmed by behavioural analysis of a heterozygous in-cross with subsequent genotyping.

### Cardiac injection of Na_2_CaEDTA

To assess the effect of Na_2_CaEDTA (disodium calcium edetate also referred to as edetate calcium disodium, ethylene diamine tetra-acetic acid disodium calcium salt) on Mn levels, s*lc39a14*^*U801*^ larvae were injected directly into the heart with 5 or 50 ng Na_2_CaEDTA after immobilization in 1.5% low-melting point agarose (in fish water with MS-222) using a pressure-pulsed Picospritzer II (General Valve Corp). Injections were performed daily at 2, 3 and 4 dpf. Following injection, embryos were removed from the agarose and raised in fish water supplemented with 50 μM MnCl_2_ from 2 dpf. Mn levels were determined from pools of 10 larvae at 5 dpf by ICP-MS as described above.

### Statistical analysis

Student's two-tailed *t*-tests using the GraphPad Prism software (version 5; GraphPad) were used to determine the statistical significance of differences between two groups. For comparisons with more than two conditions, one-way ANOVAs with Tukey's *post hoc* tests and two-way ANOVAs were determined using IBM SPSS Statistics package version 21. Probit regression analysis was performed using IBM SPSS Statistics package version 21.

## Additional information

**How to cite this article:** Tuschl, K. *et al*. Mutations in *SLC39A14* disrupt manganese homeostasis and cause childhood-onset parkinsonism–dystonia. *Nat. Commun.* 7:11601 doi: 10.1038/ncomms11601 (2016).

## Supplementary Material

Supplementary Information Supplementary Figures 1-17, Supplementary Tables 1-9 and Supplementary Note 1

Supplementary Movie 1 Subject C-II-2 prior to chelation treatment showing axial and limb hypotonia, dystonic posturing of the lower limbs, bradykinesia, hypomimia and inability to walk.

Supplementary Movie 2 Subject C-II-2 six months after commencement on regular intravenous Na2CaEDTA treatment showing significantly improved tone and posture with residual lower limb dystonia. She has regained her ability to walk with the aid of foot orthoses.

Supplementary Movie 3 Subject E-II-2 at 17 years of age, wheelchair bound, showing severe generalized and oromandibular dystonia, limb contractures and scoliosis.

Supplementary Movie 4 Subject E-II-3 at 16 years of age, wheelchair bound, showing severe generalized and oromandibular dystonia, limb contractures and scoliosis.

Supplementary Movie 5 Subject E-II-4 at 9 years of age, wheelchair bound, showing generalized dystonia, choreatic upper limb movements and limb contractures.

## Figures and Tables

**Table 1:** Clinical characteristics and *SLC39A14* mutations identified in affected patients.

**Subject**	**Gender**	**Method used to identify mutation**	***SLC39A14*** mutation*	**Amino-acid change**	**Exon**	**Isoform**	**Country of origin**	**Age of onset**	**Current age**	**Whole-blood Mn (73–325 nmol l**^**−1**^**)**
A-II-1	F	S	c.[292T>G]; [292T>G]	p.[F98V]; [F98V]	3	1–3	Yemen	7 months	13 years	2,887
A-II-2	F	S	c.[292T>G]; [292T>G]	p.[F98V]; [F98V]	3	1–3	Yemen	6 months	† (7 years)	NA
B-II-1	F	‡	‡	‡	‡	‡	Egypt	7 months	† (13 months)	NA
B-II-4	F	WES/S	c.[313G>T]; [313G>T]	p.[E105X], [E105X]	3	1–3	Egypt	7 months	3 years	8,101
C-II-2	F	S	c.[477_478del]; [477_478del]	p.[S160Cfs*5], [S160Cfs*5]	4B	2	India	3 years	6 years	963
D-II-1	M	WES/S	c.[1147G>A]; [1147G>A]	p.[G383R]; [G383R]	7	1–3	Spain	10 months	† (4 years)	965§
E-II-2	F	AM/S	c.[1407C>G]; [1407C>G]	p.[N469K]; [N469K]	9	1–3	Lebanon	2 years	17 years	2,280
E-II-3	F	AM/S	c.[1407C>G]; [1407C>G]	p.[N469K]; [N469K]	9	1–3	Lebanon	2 years	16 years	3,830
E-II-4	M	WES/AM/S	c.[1407C>G]; [1407C>G]	p.[N469K]; [N469K]	9	1–3	Lebanon	2 years	9 years	1,260

AM, autozygosity mapping; F, female; M, male; NA, not available; S, Sanger sequencing; WES, whole-exome sequencing.

Individual families are numbered A to E with each affected sibling listed.

^*^All patients were from consanguineous families and were shown to be homozygous for the mutations detected. WES, AM, S, Nucleotide (c.) and amino-acid (p.) changes refer to transcript 2 (NM_015359.4) and protein isoform 2 (NP_056174.2) and are listed together with the exon and isoform affected.

^†^Deceased, NA

^‡^DNA of this subject was not available for mutation testing. Her clinical phenotype was similar to her sibling, suggesting that they were both affected by the same disorder. Normal reference range for whole-blood Mn is given in brackets.

^§^Mn estimation performed in different hospital laboratory, reference range <145.6 nmol l^−1^.

**Table 2:** Whole-blood Mn levels are raised in SLC39A14 deficiency.

**ng ml**^**−1**^	**Control**	**F98V**	**E105X**	**Father**	**Mother**	**Reference**
Mn	6.69–11.29	*159*	*445*	15.1	12.2	5–12.8 (ref. 40)
Fe	409–462	434	397	370	386	236–614 (ref. 41)
Cu	635–1,096	1,130	944	770	1,108	590–1,470 (ref. 41)
Zn	4,364–5,284	5,076	4,580	5,804	5,424	4,800–7,800 (ref. 41)
Cd	0.35	1.42	0.18	0	0.89	0.15–2.04 (ref. 40)

Table showing whole-blood metal levels for two affected individuals (F98V and E106X), three healthy control subjects, parents heterozygous for the E105X mutation and published reference ranges^40^,^41^. While Mn levels are significantly raised in both patients, other metal levels are within the normal range. Abnormal Mn levels are indicated in italics.
