# Supplementary material for: Mutations in SLC39A14 disrupt manganese homeostasis and cause childhood-onset parkinsonism–dystonia
Source: Nat Commun. 2016 May 27;7:11601. doi: 10.1038/ncomms11601 (PMC4894980; doi:10.1038/ncomms11601)
Supplement: Supplementary Information — Supplementary Figures 1-17, Supplementary Tables 1-9 and Supplementary Note 1 [file ncomms11601-s1.pdf]

Supplementary Figures

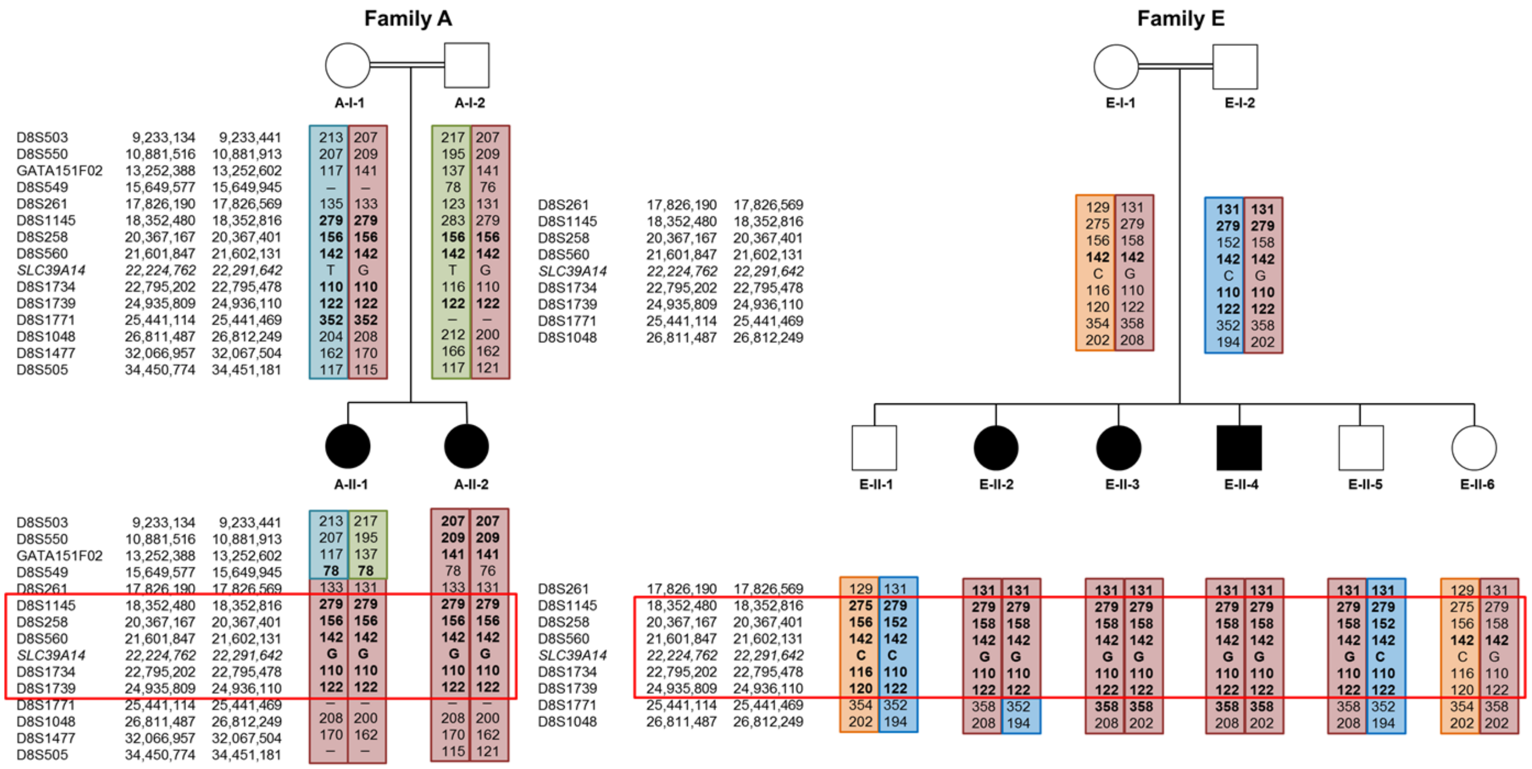

Supplementary Figure 1. Microsatellite marker linkage analysis on chromosome 8 in Family A and Family E. *SLC39A14* is located at 8p21.3. Microsatellite markers are positioned according to physical distance (measured in Mb; localisation according to NCBI build 36.3). Haplotypes for these markers are shown, and the disease-associated haplotypes are boxed in pink. Green, blue, and orange boxes indicate non–disease associated haplotypes. All affected children of each family share a common homozygous haplotype in this region (indicated by the pink boxes).

```
Human            MKLLLLHPAFQSCLLLTLLGLWRTTPEAHASSLGAPAISAASFLQDLIHRYGEGDSLTLQ 60
Chimpanzee       --MKLLHPAFQSCLLLTLLGLWRTTPEAHASSPGAPAISAASFLQDLIHRYGEGDSLTLQ 58
Mouse            --MKRLHPALPSCLLLVLFGIWRTAPQTHASSAGLPPLSATSFLEDLMDRYGKNDSLTLT 58
Chicken          -------MANPAVLLLLLSLRWVPA----GSDAVLPQLSAASFVQDLLRRYGGGEALSLE 49
Zebrafish        MTLRRASGCRQLTLTIGLALTLGLLQWPIGDVRGQDGASPAQVLQELLTRYGDNASISVP 60
                         .    * : *           ..       *.:..:::*: *** . ::::

Human            QLKALLNHLDVGVGRGNVTQHVQGHRNLSTCFSSGDLFTAHNFSEQSRIGSSELQEFCPT 120
Chimpanzee       QLKALLNHLDVGVGRGNVTQHVQGHRNLSTCFSSGDLFTAHNFSEQSRIGSSELQEFCPT 118
Mouse            QLKSLLDHLHVGVGRDNVSQPKEGPRNLSTCFSSGDLFAAHNLSERSQIGASEFQEFCPT 118
Chicken          QLKALLNRLDVGVGRSNGSRP---HANLSRCFSSAELFAVHNLSEGSVLGAAELRAFCPA 106
Zebrafish        QLRSLLVRLNGGQSEDHDSKTQPTRTNASKCLA-ADTLAVYGMSEQSRIDERGLQQICPT 119
                 **::** :*. * ...: ::      * * *:: .: ::.:.:** * :.   :: :**:

Human            ILQQLDSRACTSENQENEENEQTEEGRPSAVEVWGFGFLSVSLINLASLLGVLVLPCTEK 180
Chimpanzee       ILQQLDSRACTSENQENEENEQTEEGRPSAVEVWGYGLLCVTIISLCSLLGASVVPFKKK 178
Mouse            ILQQLDSQACTSENQKSEENEQTEEGKPSAIEVWGYGFLCVTVISLCSLMGASVVPFMKK 178
Chicken          VLQQLESAACAAENLENEENEQTEEGRPSAAEVWGFGFLSVSMINVASLLGLLIVPCTRK 166
Zebrafish        MIQQLDSQACKTQ--PNQESESSP--RPTEAEVWGYSILSVTLVSAFALTGVFVVPLMRT 175
                 ::***:* ** ::   .:*.*.:   :*:  ****:.:*.*:::.  :* *  ::*  ..

Human            AFFSRVLTYFIALSIGTLLSNALFQLIPEAFGFNPLEDYYVSKSAVVFGGFYLFFFTEKI 240
Chimpanzee       TFYKRLLLYFIALAIGTLYSNALFQLIPEAFGFNPLEDYYVSKSAVVFGGFYLFFFTEKI 238
Mouse            TFYKRLLLYFIALAIGTLYSNALFQLIPEAFGFNP-QDNYVSKSAVVFGGFYLFFFTEKI 237
Chicken          AFFSRILTFFIALSIGTLLSNALFQLIPEAFGFNPQEDYYVSKSAVVFGGFYLFFFTEKI 226
Zebrafish        RFMRRVLVYFIALSIGTLLSTAILQLLPEAFGFDPMEDYYVPKSAVVFGGFYLFFFTEKI 235
                  *  *:* :****:**** *.*::**:******:* :* **.******************

Human            LKILLKQKNE--HHHGHSHYASESLP-SKKDQEEGVMEKLQNGDLD-HMIPQHCSSELDG 296
Chimpanzee       LKILLKQKNE--HHHGHSHYASESLP-SKKDQEEGVMEKLQNGDLD-HMIPQHCSSELDG 294
Mouse            LKMLLKQKNE--HHHGHNHFTSETLP-SKKDQEEGVTEKLQNGDLD-HMIPQHCNSELDG 293
Chicken          LKMLLKQKDP--HHHGHSHYSTEALP-SRKDREEGVTEKLQNGDLD-HMIP-HVANEMEC 281
Zebrafish        LKMILKPKDTGGHGHGHSHFPAERYANSNGDLEDGVMEKLQNGEAGGAALP---RAEADG 292
                 **::** *:   * ***.*:.:*  . *. * *:** ******: .   :*     * :

Human            KAPMVDEKVIVGSLSVQDLQAS----QSACYWLKGVRYSDIGTLAWMITLSDGLHNFIDG 352
Chimpanzee       KAPMVDEKVIVGSLSVQDLQAS----QSACYWLKGVRYSDIGTLAWMITLSDGLHNFIDG 350
Mouse            KAPGTDEKVIVNSMSVQDLQAS----QSACYWLKGVRYSDIGTLAWMITLSDGLHNFIDG 349
Chicken          KSPPGDEKVVVGSLSVQDLQAS----QSACYWLKEVRYSDIGTLAWMITLSDGLHNFIDG 337
Zebrafish        RGVGEDDKMLSTGQTVQDTQSSGGGGTGGCYWLKGRAYSDIGTLAWMITLSDGLHNFIDG 352
                 :.   *:*::  . :*** *:*     ..*****   ***********************

Human            LAIGASFTVSVFQGISTSVAILCEEFPHELGDFVILLNAGMSIQQALFFNFLSACCCYLG 412
Chimpanzee       LAIGASFTVSVFQGISTSVAILCEEFPHELGDFVILLNAGMSIQQALFFNFLSACCCYLG 410
Mouse            LAIGASFTVSVFQGISTSVAILCEEFPHELGDFVILLNAGMSIQQALFFNFLSACCCYLG 409
Chicken          LAIGASFTVSVFQGISTSVAILCEEFPHELGDFVILLNAGMTIRQALFFNFISACCCYVG 397
Zebrafish        LAIGASFTASVFQGISTSVAILCEEFPHELGDFVILLNAGMSIQQALFFNFLSACCCYLG 412
                 ********.********************************:*:*******:******:*

Human            LAFGILAGSHFSANWIFALAGGMFLYISLADMFPEMNEVCQEDERKGS--ILIPFIIQNL 470
Chimpanzee       LAFGILAGSHFSANWIFALAGGMFLYISLADMFPEMNEVCQEDERKGS--ILIPFVIQNL 468
Mouse            LAFGILAGSHFSANWIFALAGGMFLYIALADMFPEMNEVCQEDEKNDS--FLVPFVIQNL 467
Chicken          LAFGIVAGSHFSANWIFALAGGMFLYIALADMFPEMNEVSREDEQNGS--ALITFAIQNA 455
Zebrafish        MGFGILAGNNFSPNWIFALAGGMFLYIALADMFPEMNEVSREEEEAGGSGFLLTFALQNA 472
                 :.***:**.:**.**************:***********.:*:*. ..   *:.* :**

Human            GLLTGFTIMVVLTMYSGQIQIG 492
Chimpanzee       GLLTGFTIMVVLTMYSGQIQIG 490
Mouse            GLLTGFSIMLVLTMYSGQIQIG 489
Chicken          GLLTGFTIMVLLTMYSGQIQIG 477
Zebrafish        GLLTGFAIMLVLTIYSGQIQLG 494
                 ******:**::**:******:*
```

Supplementary Figure 2. Evolutionary conservation data for SLC39A14 Isoform 2. ClustalW software (http://www.ebi.ac.uk/Tools/msa/clustalw2/) was used to generate the alignment. Residues identical to the human *SLC39A14* sequence are marked with an asterisk (*). Conservation between amino acids of strongly and weakly similar properties is indicated by a colon (:) and a period (.), respectively. Putative protein domains are highlighted and include a signalling peptide (position 1-30; purple; numbering according to the human protein

sequence), eight transmembrane domains (position 156-177, 186-211, 225-245, 337-359, 365-389, 397-420, 427-450, 465-487; grey), a histidine-rich region (position 251-257, turquoise), and the LZT consensus motif (375-384; turquoise). Amino acids affected by missense mutations are highlighted in yellow (F98V, G383R, N469K) and those pertaining to truncating mutations in green (E105X, S160Cfs*5). The site of the CRISPR induced mutation in zebrafish is indicated in red (P210Hfs*48). Protein sequences used to generate this alignment are NP_056174.2 (human), XP_531112.3 (chimpanzee), NP_659057.2 (mouse), XP_427108.3 (chicken), and XP_005171823.1 (zebrafish).

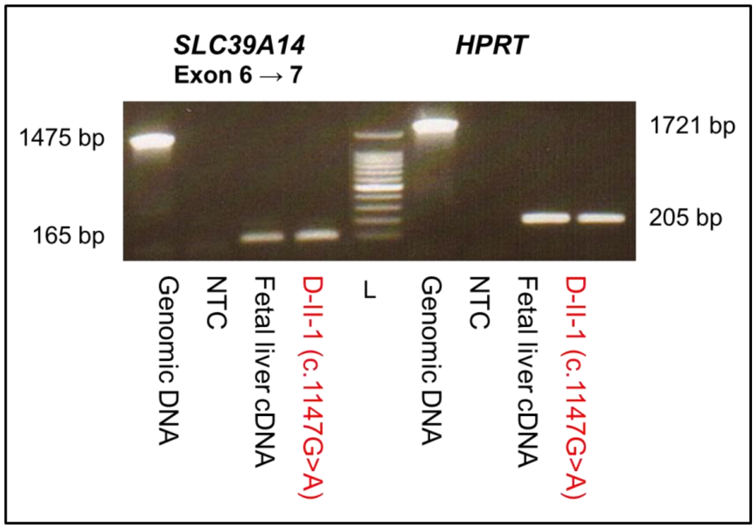

Supplementary Figure 3. The c.1147G>A mutation does not affect normal splicing. RT-PCR on liver cDNA from individual D-II-1 produces a single band of 165 bp confirming normal mRNA splicing. Fetal liver, shown to express *SLC39A14* (**Fig. 2c**), was used as a control sample. Primers used span *SLC39A14* exon 6 (5' GTGCTTCCTTCACTGTGTCA 3') and exon 7 (5' CGTTGAGCAGGATGACAAAG 3'). Genomic and unspliced cDNA are predicted to generate a 1475 bp amplicon, whilst normally spliced cDNA produces a 165 bp amplicon. Hypoxanthine-guanine phosphoribosyltransferase (HPRT) was used as a housekeeping control gene. L, 100 bp ladder (Promega). NTC, non-template control.

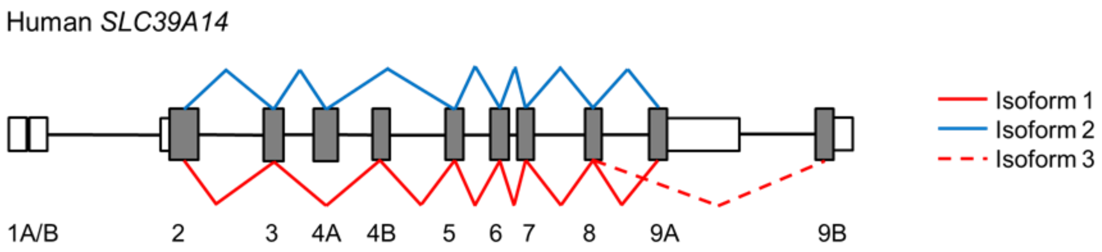

Supplementary Figure 4. Schematic of the human *SLC39A14* gene. *SLC39A14* is composed of nine exons, two of which (exon 4 and 9) can be alternatively spliced to generate three isoforms. Isoform 1 (NP_001121903) is encoded by two different transcripts that have an alternative 5'UTR (NM_001128431.2 and NM_001135153.1). Isoform 2 (NP_056174) is encoded by one transcript only (NM_015359.4). Isoform 1 and 2 span 492 amino acids and differ by 20 amino acids encoded by an alternatively spliced exon four (4A and 4B). Transcript NM_001135154.1 which encodes isoform 3 (NP_001128626) has an alternative exon nine but shares the remaining protein sequence with isoform 1. Coding exons in grey, untranslated regions unshaded (not drawn to scale).

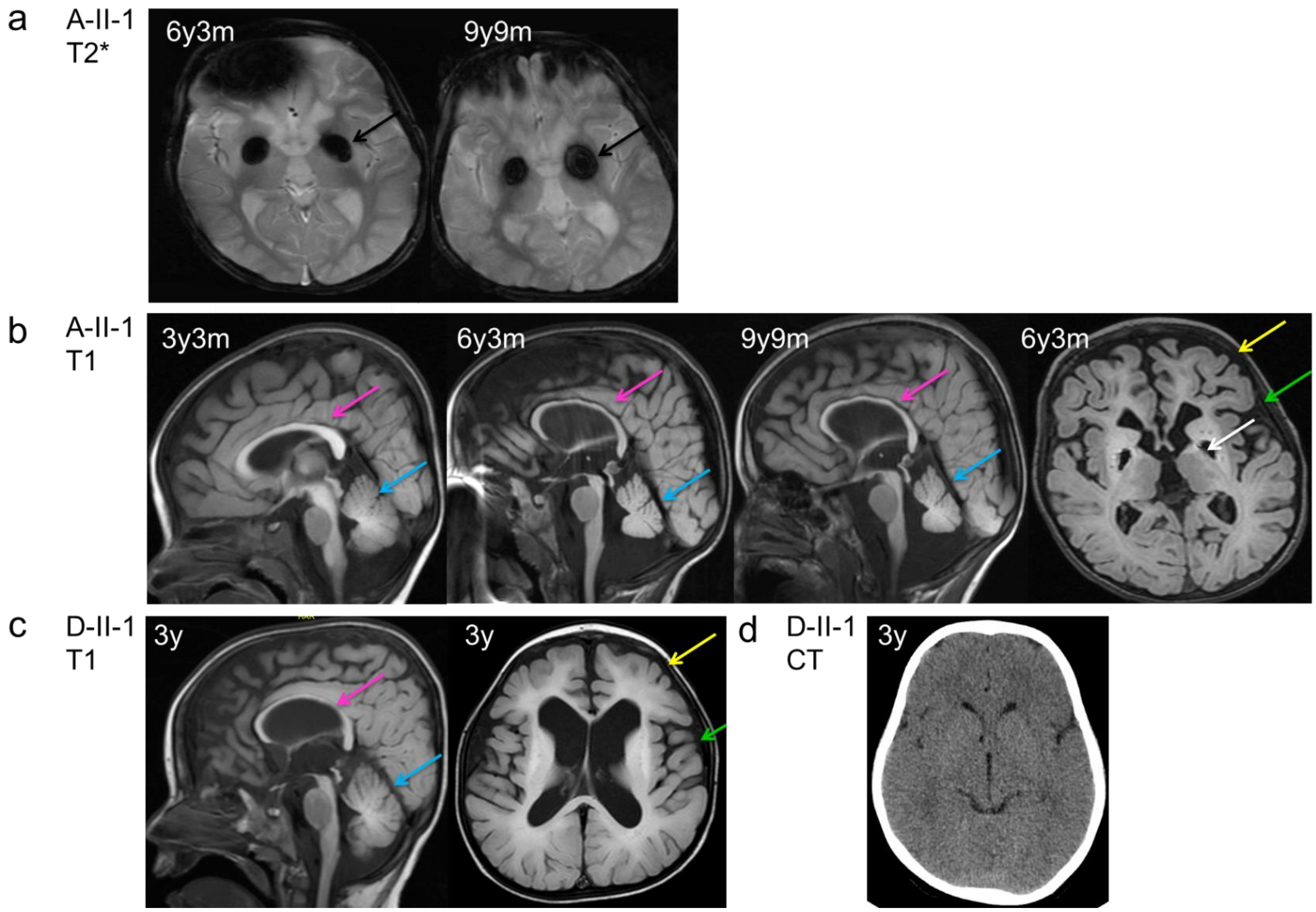

Supplementary Figure 5. Additional MRI brain features of individuals with mutations in *SLC39A14*. (**a**) Serial T2-weighted imaging of individual A-II-1 shows progressive signal hypointensity within the globus pallidus over time (black arrow). (**b**) and (**c**) Some patients develop cortical atrophy (yellow arrows), cerebellar atrophy (blue arrows) with thinning of the corpus callosum (pink arrows) and widening of the extracerebral spaces (green arrows). Note hyperintense signal within the basal ganglia associated with central pallidal signal hypointensity due to extreme T2 effects dominating the central region of the pallidum on the T1-weighted image (white arrow). (**d**) CT brain imaging of individual D-II-1 is normal.

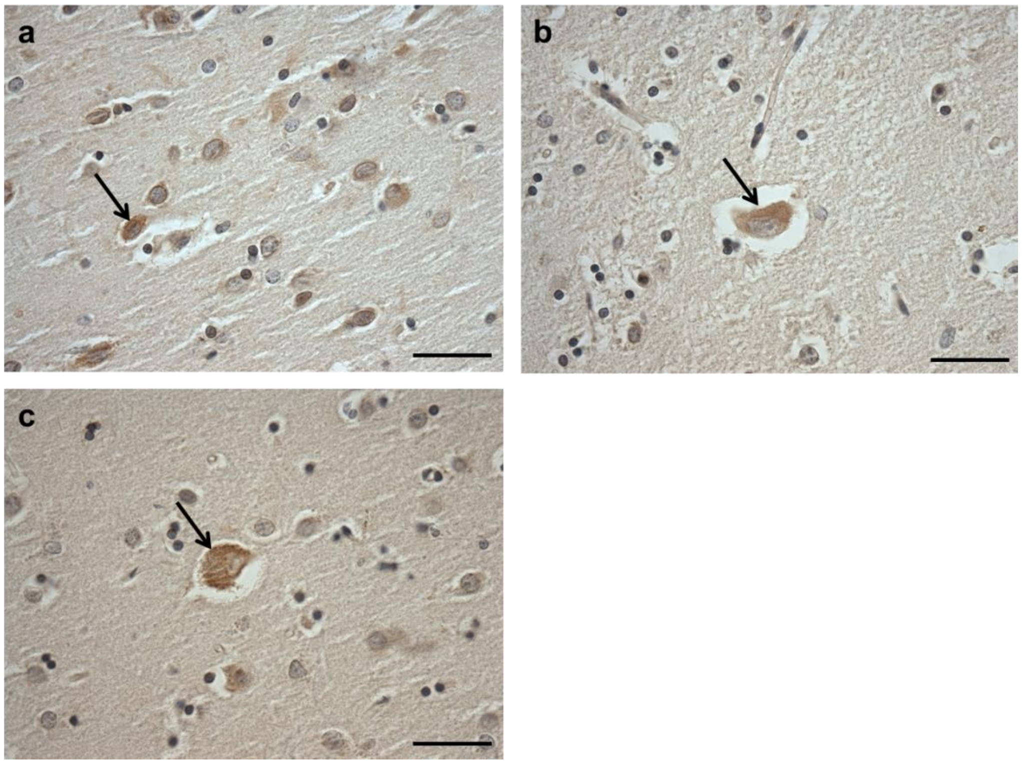

Supplementary Figure 6. SLC39A14 is expressed in large neurons of the insular cortex, the caudate nucleus and putamen. Immunostaining for human SLC39A14 demonstrates expression in neurons of the (a) insular cortex, (b) caudate nucleus, (c) putamen. Arrows pointing at positively stained neurons. Rabbit polyclonal anti-SLC39A14 antibody (1:1000, NBP1-81551, Novus). Scale bar 100 µm.

```
Exon4A      TGTGGGGCTTTGGTTTTCTCAGTGTCTCACTGATTAACCTGGCCTCTCTCCTGGGAGTCC 60
Exon4B      TGTGGGGATACGGTCTCCTCTGTGTGACCGTCATCTCCCTCTGCTCCCTCCTGGGGGCCA 60
            ******* *  *** * *** ****  *  * **   ***   *** ******** * *

Exon4A      TCGTCCTGCCCTGCACAGAGAAAGCGTTTTTCAGCCGTGTGCTCACTTACTTCATCGCCC 120
Exon4B      GCGTGGTGCCCTTCATGAAGAAGACCTTTTACAAGAGGCTGCTGCTCTACTTCATAGCTC 120
             ***  ****** **   ****  * **** **   *  ****    ******** ** *

Exon4A      TGTCCATTGGAACGCTGCTGTCTAACGCGCTATTCCAGCTCATCCCAGAG 170
Exon4B      TGGCGATTGGAACCCTCTACTCCAACGCCCTCTTCCAGCTCATCCCGGAG 170
            ** * ******** **    ** ***** ** ************** ***
```

Supplementary Figure 7. Alignment of human *SLC39A14* exon 4A and 4B. Nucleotide sequences used to generate this alignment are NM_015359.4 (exon 4A) encoding isoform 2 and NM_001128431.2 (exon 4B) encoding isoform 1. Nucleotides differing between the two exons are highlighted in yellow. Primers used for RT-PCR are underlined (**Supplementary Table 3**).

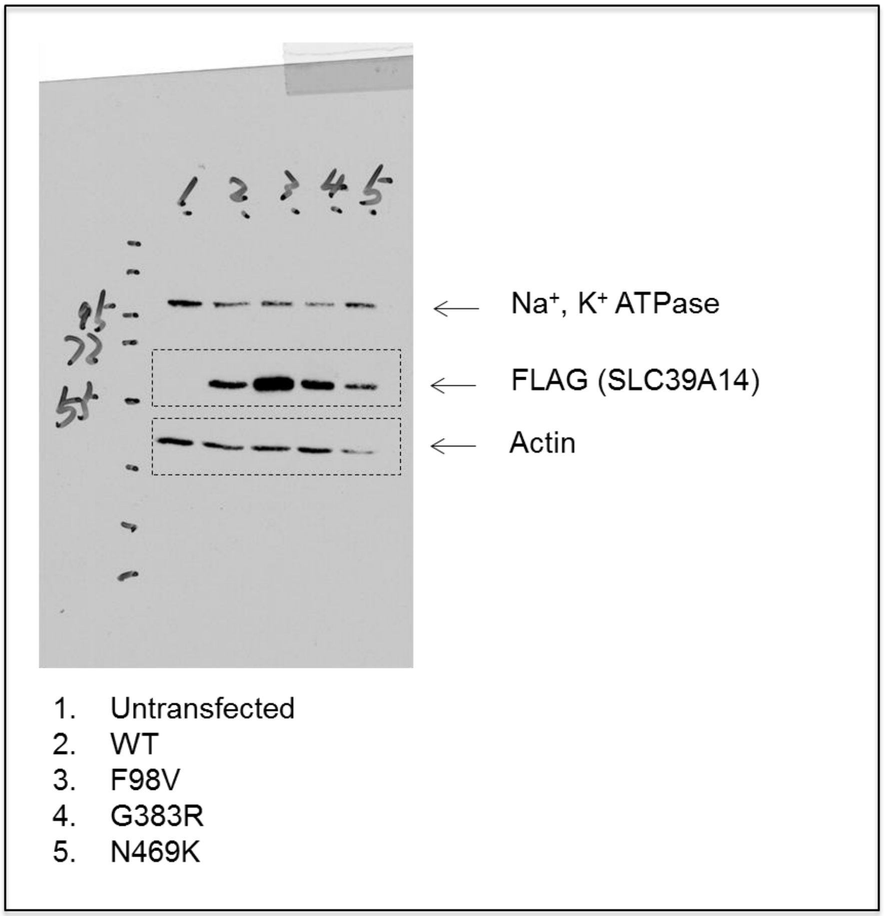

Supplementary Figure 8. Full length immunoblot of whole cell lysates of stably transfected HEK-293 cells showing expression of the wild-type and three mutant SLC39A14 proteins. Actin and Na+, K+ ATPase were used as a loading control. The blot was first incubated with mouse anti-FLAG antibody to detect SLC39A14 followed by incubation with mouse anti-Actin antibody and mouse anti Na+, K+ ATPase. Areas of the cropped image are indicated by dashed boxes.

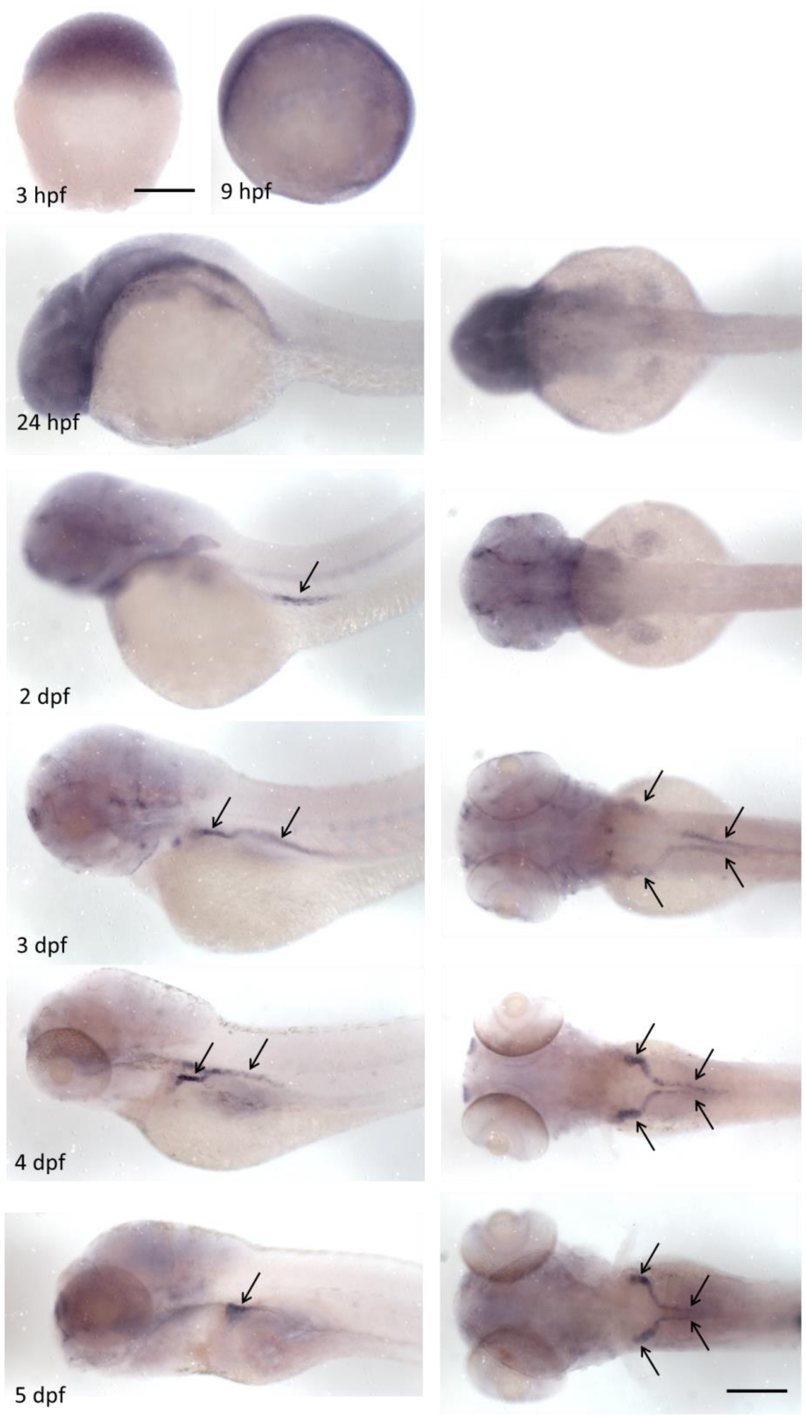

Supplementary Figure 9. Time course of *slc39a14* expression during embryonic and early larval development in zebrafish. Whole mount *in situ* hybridisation using a DIG-labelled anti-sense RNA probe shows prominent expression of *slc39a14* in the proximal convoluted and straight pronephric tubules (arrows) from 2 dpf. Scale bar 200 µm.

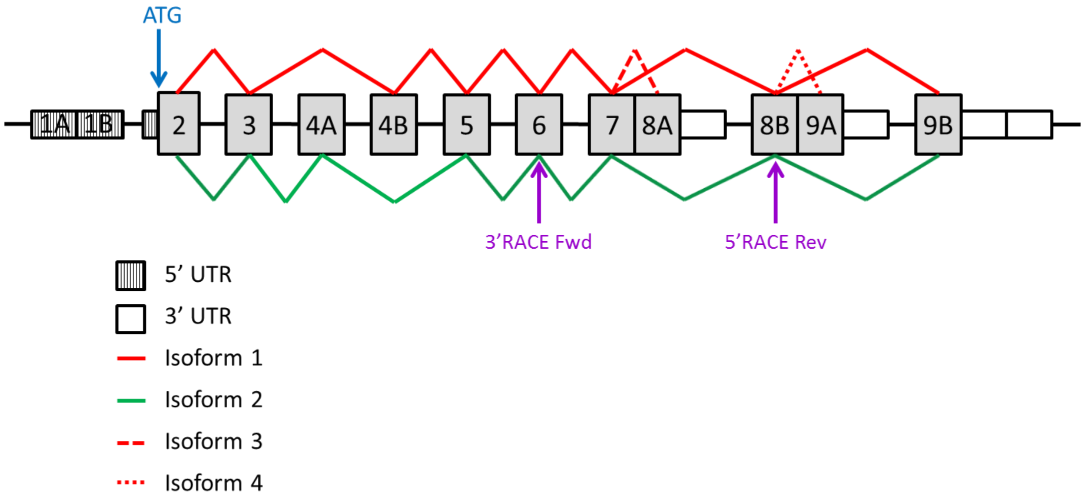

Supplementary Figure 10. Schematic of zebrafish *slc39a14*. 5' and 3' RACE using RNA extracted from zebrafish larvae at 3 dpf identified four alternative transcripts. Isoform 1 (red) and 2 (blue) are encoded by an alternatively spliced exon four. Isoform 3 (dotted red) is encoded by an alternative ninth exon and 3' UTR. Isoform 4 (dashed red) is encoded by a shorter transcript with only eight exons (not drawn to scale). The ATG start codon is marked in blue, gene specific inner primers used for 5' and 3' RACE are marked in purple.

```
HS MKLLLLHPAFQSCLLLTLLGLWRTTPEAHASSLGAPAISAASFLQDLIHRYGEGDSLTLQ 60
DR MTLRRASGCRQLTLTIGLALTLGLLQWPIGDVRGQDGASPAQVLQELLTRYGDNASISVP 60
   *.*     . *  * : *         . ..  *  . *.*..**:*: ***:. *:::

HS QLKALLNHLDVGVGRGNVTQHVQGHRNLSTCFSSGDLFTAHNFSEQSRIGSSELQEFCPT 120
DR QLRSLLVRLNGGQSEDHDSKTQPTRTNASKCLAA-DTLAVYGMSEQSRIDERGLQQICPT 119
   **::** *:* * ...: ::    : * *.*::: * ::.:.:******..  **::***

HS ILQQLDSRACTSENQENEENEQTEEGRPSAVEVWGYGLLCVTVISLCSLLGASVVPFMKK 180
DR MIQQLDSQACKT--QPNQESESSP--RPTEAEVWGYGLLCVTVISLCSLVGASVVPFMRK 175
   ::*****:**.:  * *:*.*.:   **: .******************:********:*

HS TFYKRLLLYFIALAIGTLYSNALFQLIPEAFGFNPLEDYYVSKSAVVFGGFYLFFFTEKI 240
DR TFYKRLLLYFIALAIGTLYSNALFQLIPEAFGFDPMEDYYVPKSAVVFGGFYLFFFTEKI 235
   *********************************:*:*****.******************

HS LKILLKQKNE--HHHGHSHYASESLP-SKKDQEEGVMEKLQNGDLDHMIPQHCSSELDGK 297
DR LKMILKPKDTGGHGHGHSHFPAERYANSNGDLEDGVMEKLQNGEAGGAALP--RAEADGR 293
   **::** *:   * *****:.:*  . *: * *:*********: .        :* **:

HS APMVDEKVIVGSLSVQDLQAS----QSACYWLKGVRYSDIGTLAWMITLSDGLHNFIDGL 353
DR GVGEDDKMLSTGQTVQDTQSSGGGGTGGCYWLKGRAYSDIGTLAWMITLSDGLHNFIDGL 353
   .   *:*::  . :*** *:*     ..******  ************************

HS AIGASFTVSVFQGISTSVAILCEEFPHELGDFVILLNAGMSIQQALFFNFLSACCCYLGL 413
DR AIGASFTASVFQGISTSVAILCEEFPHELGDFVILLNAGMSIQQALFFNFLSACCCYLGM 413
   *******.***************************************************:

HS AFGILAGSHFSANWIFALAGGMFLYISLADMFPEMNEVCQEDERKGS--ILIPFIIQNLG 471
DR GFGILAGNNFSPNWIFALAGGMFLYIALADMFPEMNEVSREEEEAGGSGFLLTFALQNAG 473
   .******.:**.**************:***********.:*:*. *.  :*:.* :** *

HS LLTGFTIMVVLTMYSGQIQIG 492
DR LLTGFAIMLVLTIYSGQIQLG 494
   *****:**:***:******:*
```

Supplementary Figure 11. Zebrafish Slc39a14 shares 62 percent sequence identity with human SLC39A14. Sequence alignment of human (top) and zebrafish (bottom) SLC39A14 protein sequence. Protein sequences of human isoform 1 (NP_001121903.1) and zebrafish isoform 1 (XP_001340102.4) were aligned using ClustalW2 (http://www.ebi.ac.uk/Tools/msa/clustalw2/). Residues identical to the human SLC39A14 sequence are marked in yellow with an asterisk (*). Conservation between amino acids of strongly and weakly similar properties is indicated by a colon (:, dark grey) and a period (. light grey), respectively. The putative transmembrane domains are underlined. The histidine-rich region and the LZT consensus motif are highlighted in red. The position of the CRISPR induced deletion is indicated in turquoise. HS, homo sapiens; DR, danio rerio.

a

F0

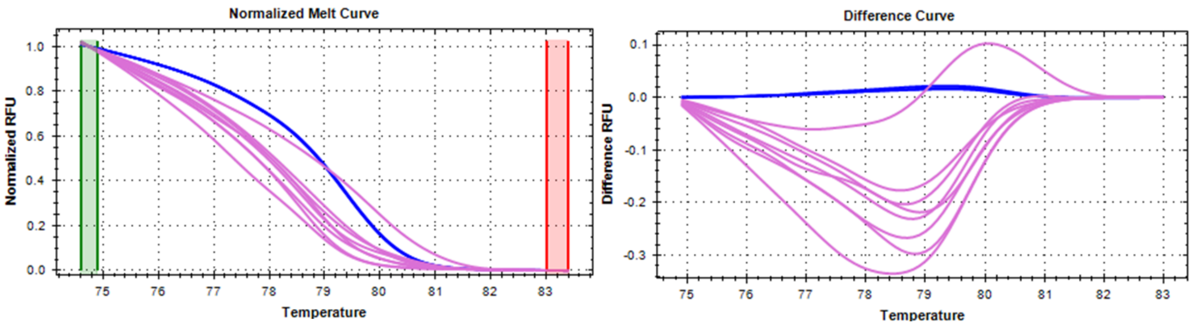

b

F1

```
cctgtatgtagGCCTTCGGGTTTGACCCCATGGAGGATTATTATGTGCCCAA WT

cctgtatgtagGCCTTCGGGTTTGACCC-ATGGAGGATTATTATGTGCCCAA Δ1
cctgtatgtagGCCTTCGGGTTTGACC--ATGGAGGATTATTATGTGCCCAA Δ2
cctgtatgtagGCCTTCGGGTTT----CCATGGAGGATTATTATGTGCCCAA Δ4
cctgtatgtagGCCTTCGGGTTT---------GAGGATTATTATGTGCCCAA Δ9
cctgtatgtagGCCTTCGGGTTTGA---------------TTATGTGCCCAA Δ15
cctgtatgtagGCCTTCGGGTTTGAGAGGATTAATAATCCATGGAGGATTAT Δ2, Ins13
```

slc39a14^{U801} mutant: c.629_630del, p.P210Hfs*48

Supplementary Figure 12. CRISPR induced mutagenesis of zebrafish *slc39a14*. (**a**) High Resolution Melting Analysis (HRMA) shows that co-injection of a gRNA to exon 5 (target sequence 5' GGCCTTCGGGTTTGACCCCA 3') and Cas9 mRNA results in a high somatic mutagenesis rate (>90%) in zebrafish embryos at 1 dpf (F0 generation). Normalized melt curve (left) and difference curve (right) show differences in the melt curve shape between un-injected wild-type (blue) and (purple) injected embryos. The green and red boxes indicate stable pre- and post-melt fluorescence intensity used for data normalisation. RFU, relative fluorescence units. (**b**) CRISPR induced indel mutations identified in the F1 generation. Wild-type (WT) sequence given in the top row. gRNA target sequence highlighted in yellow; insertions in grey; -, single nucleotide deletion; Δ, number of deleted base-pairs. The *slc39a14*^{U801} mutant is highlighted by a red box.

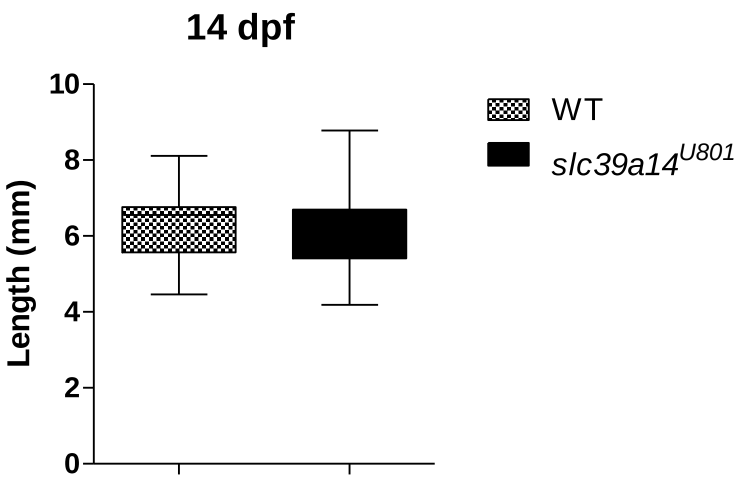

Supplementary Figure 13. *Slc39a14^{U801}* mutants do not show differences in size compared to wild-types. Plots of the larval length of wild-type (WT, n=56) and *slc39a14^{U801}* (n=64) zebrafish at 14 dpf show no significant difference. The box and whisker plots indicate the 25th and 75th percentile, and the minimum and maximum length, respectively. Statistical analysis was performed using Student's *t* test (p=0.062).

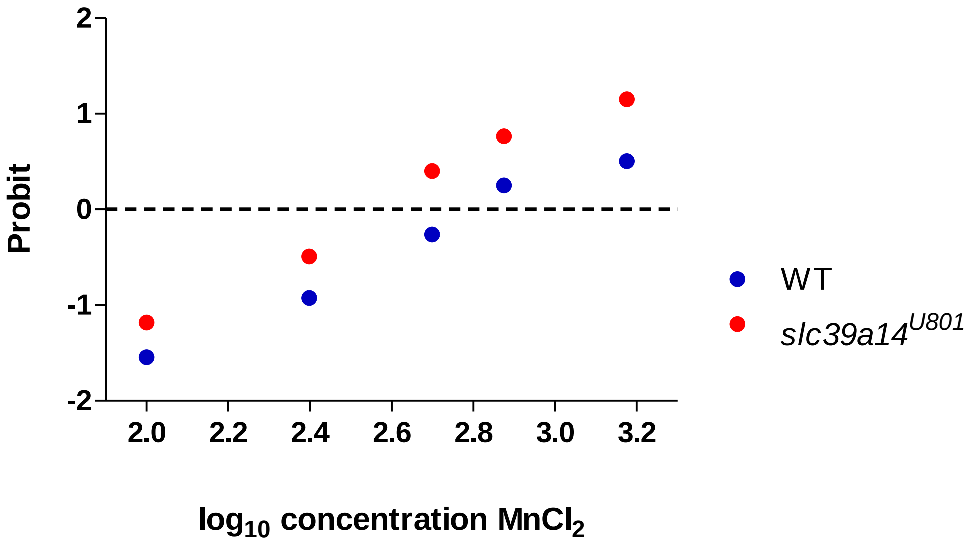

Supplementary Figure 14. *Homozygous slc39a14^{U801} mutants are more sensitive to $MnCl_2$ toxicity compared to wild-type larvae.* Graph showing the dose-response curves for wild-type (red) and mutant (blue) larvae determined by Probit analysis using IBM SPSS Statistics package version 21. The logarithmic concentration of $MnCl_2$ (x-axis) is plotted against the determined Probit (probability unit, y-axis). The Probit of 0 is used to determine the LC_{50} concentration.

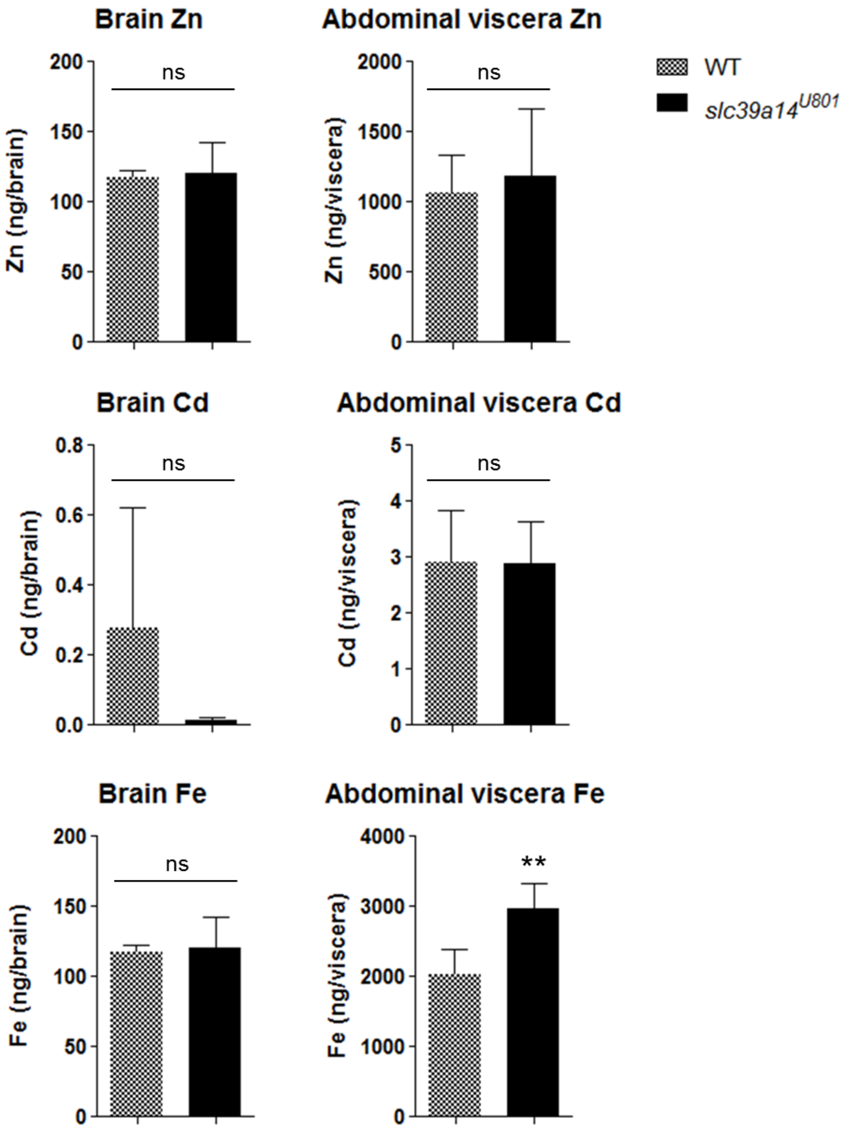

Supplementary Figure 15. Metal levels in adult tissues of wild-type and homozygous *slc39a14*^{U801} zebrafish. There is no difference in Zn and Cd levels between s*lc39a14*^{U801} mutants and wild-types (WT). While Fe levels are not altered in the brain (p=0.74 [Zn], p=0.4 [Cd], p=0.88 [Fe]), there is an increase in Fe concentration in abdominal viscera of mutant fish (p=0.66 [Zn], p=0.96 [Cd], p=0.0037 [Fe]). Measurements were taken from pools of four brains / abdominal viscera. Data are presented as means ± s.d. from two independent experiments. Statistical analysis was performed using Student’s two tailed *t* test (**p<0.01; ns, not significant).

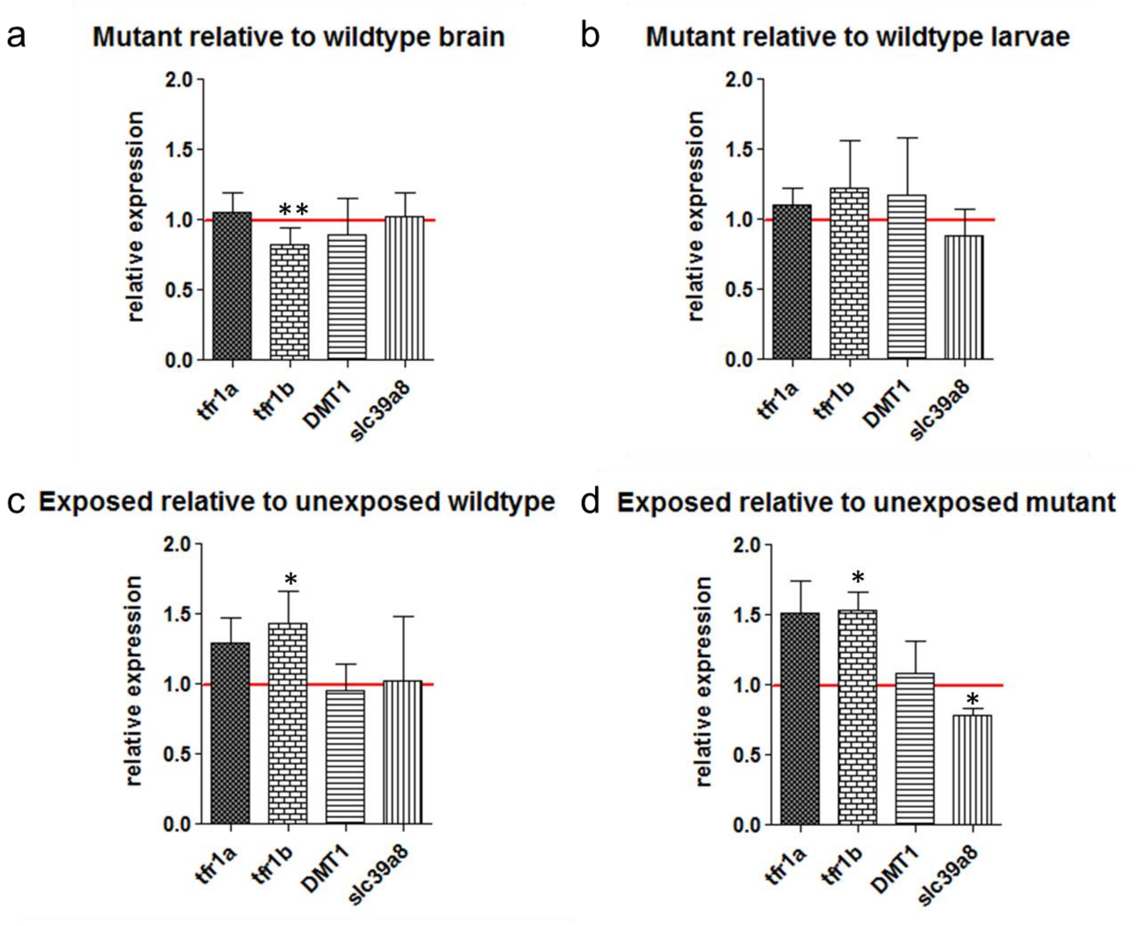

Supplementary Figure 16. Graph showing Mn uptake transporter gene expression (*tfr1a*, *tfr1b*, *DMT1*, *slc39a8*) in homozygous *slc39a14*^{U801} mutants relative to wild-type zebrafish. (a**)** *tfr1b* expression is reduced in mutant brain (p=0.005) while there is no change for *tfr1a*, *DMT1* and *slc39a8* expression (p=0.83/0.36/0.89). (**b**) No changes in transporter transcript levels are seen in mutant relative to wild-type larvae at 5 dpf (p=0.72/0.35/0.41/0.072). (**c**) and (**d**) Mn exposure (50µM $MnCl_2$ from 2 to 5 dpf) of zebrafish larvae leads to upregulation of *tfr1b* expression in both wild-type (p=0.042) and mutant zebrafish (p=0.038) at 5 dpf while *slc39a8* transcript levels are reduced in mutant larvae only (p=0.049). Data are presented as means ± s.d. from three independent experiments. Statistical analysis was performed using Student's two tailed *t* test on individual ∆Ct values (*p<0.05, **p<0.01).

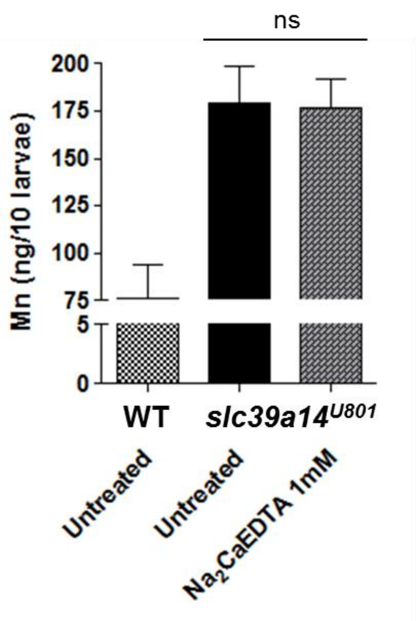

Supplementary Figure 17. Immersion of *slc39a14^{U801}* larvae in $Na_2CaEDTA$ does not reduce Mn levels. Graph shows Mn levels of wild-type (WT) and *slc39a14^{U801}* larvae exposed to 50 µM $MnCl_2$ from 2 to 5 dpf. At 5 dpf larvae were transferred to standard fish water without addition of $MnCl_2$. *slc39a14^{U801}* larvae were supplemented with 1mM $Na_2CaEDTA$ between 5 and 7 dpf. Mn levels measured in pools of 10 larvae at 7 dpf did not show differences between $Na_2CaEDTA$ treated and untreated larvae. Data are presented as means ± s.d. from three independent experiments. Statistical analysis was performed using one way ANOVA and Tukey multiple comparisons test (ns, not significant; $p=0.98$).

Supplementary Tables

Gene	Locus	Phenotype	Changes	DNA level	Protein level	MAF	ExAC	Status	PolyPhen2	SIFT	PROVEAN
**Patient B-II-4**											
SCNA	PARK1/ PARK4	PD 1/PD 4	none found	–	–	–	–	–	–	–	–
PARK2	PARK2	PD, juvenile, type 2	none found	–	–	–	–	–	–	–	–
PINK1	PARK6	PD 6, early onset	rs2298298	c.388-7A>G	NA	0.18(A)	0.862(G)	hom	NA	NA	NA
			rs3131713	c.960-5G>A	NA	0.18(G)	0.863(A)	hom	NA	NA	NA
			rs686658	c.*37A>T	NA	0.16(A)	0.136(A)	hom	NA	NA	NA
PARK7	PARK7	PD 7, autosomal recessive early-onset	rs226249	c.-99T>C	NA	0.36(T)	–	hom	NA	NA	NA
LRRK2	PARK8	PD 8	rs2256408	c.149G>A	p.Arg50His	0.03(G)	0.991(A)	hom	B(0.000)	T(1.000)	N(0.94)
			rs11564148	c.4939T>A	p.Ser1647Thr	0.29(A)	0.298(A)	hom	B(0.000)	T(0.953)	N(0.15)
			rs3761863	c.7190T>C	p.Met2397Thr	0.45(T)	0.376(T)	hom	B(0.000)	T(0.466)	N(-0.53)
ATP13A2	PARK9	Kufor-Rakeb syndrome	rs7531163	c.3084-3C>T	NA	0.31(A)	0.215(A)	het	NA	NA	NA
TAF1	PARK12	Dystonia-Parkinsonism, X-linked	none found	–	–	–	–	–	–	–	–
FBX07	PARK15	PD 15, autosomal recessive	none found	–	–	–	–	–	–	–	–
VPS35	PARK17	PD 17	none found	–	–	–	–	–	–	–	–
DNAJC6	PARK19	PD 19, juvenile-onset	none found	–	–	–	–	–	–	–	–
SYNJ1	PARK20	PD 20, early-onset	none found	–	–	–	–	–	–	–	–
PRKRA	DYT16	Dystonia 16	rs3997876	c.*3C>T	NA	–	0.241(A)	het	NA	NA	NA
			rs77419724	c.677T>A	p.Ile226Asn	–	0.163(T)	het	PD(0.996)	D(0.013)	D(-4.72)
			rs75862065	c.380C>T	p.Pro127Leu	–	0.098(A)	het	PD(1.000)	D(0.01)	D(-8.60)
			rs61999302	c.173A>G	p.Asp58Gly	–	0.043(C)	het	B(0.367)	D(0.172)	D(-2.48)
			rs62176112	c.32C>T	p.Pro11Leu	0.0457(A)	0.125(A)	het	B(0.001)	T(0.134)	N(0.72)
			rs141354030	c.22_23delGC	p.Ala8Argfs	0.238(G)	0.364(GC)	het	NA	NA	NA
			rs9406386	c.1A>C	p.Met1Leu	–	0.462(G)	het	B(0.000)	D(0.000)	N(-0.77)
			rs62176113	c.-24G>A	NA	–	0.276(C)	het	NA	NA	NA
			rs80197731	c.-133C>G	NA	–	–	het	NA	NA	NA
			rs797021523	c.-146C>G	NA	–	–	het	NA	NA	NA
SLC6A3	–	Parkinsonism-dystonia, infantile	none found	–	–	–	–	–	–	–	–
SLC30A10	–	Hypermanganesemia with dystonia, polycythemia, and cirrhosis	none found	–	–	–	–	–	–	–	–
SLC39A8	–	Hypomanganesemia, CDG IIn	rs151371	c.*270G>A	NA	0.499(T)	–	het	NA	NA	NA
			rs11382393	c.*262_263insC	NA	0.23((G)	–	hom	NA	NA	NA
			rs35608353	c.*258G>A	NA	0.498(T)	–	het	NA	NA	NA
			rs151370	c.*88T>C	NA	0.424(A)	–	het	NA	NA	NA
**Patient D-II-1**											
SCNA	PARK1/ PARK4	PD 1/PD 4	none found	–	–	–	–	–	–	–	–
PARK2	PARK2	PD, juvenile, type 2	rs1801334	c.1180G>A	p.Asp394Asn	0.01(T)	0.026(T)	het	PD(0.556)	PD(0.077)	D(-3.35)

PINK1	PARK6	PD 6, early onset	rs2298298	c.388-7A>G	NA	0.18(A)	0.862(G)	hom	NA	NA	NA
			rs3131713	c.960-5G>A	NA	0.18(G)	0.863(A)	hom	NA	NA	NA
			rs1043424	c.1562A>C	p.Asn521Thr	0.30(C)	0.297(C)	het	B(0.022)	T(0.247)	N(-0.94)
			rs686658	c.*37A>T	NA	0.17(A)	0.136(A)	hom	NA	NA	NA
			rs513414	c.*181C>G	NA	0.16(C)	–	hom	NA	NA	NA
			rs1043443	c.*196T>C	NA	0.30(C)	–	het	NA	NA	NA
PARK7	PARK7	PD 7, autosomal recessive early-onset	none found	–	–	–	–	–	–	–	–
LRRK2	PARK8	PD 8	rs2256408	c.149G>A	p.Arg50His	0.03(G)	0.991(A)	hom	B(0.000)	T(1.000)	N(0.94)
ATP13A2	PARK9	Kufor-Rakeb syndrome	rs15786	c.*124C>T;	NA	0.01(A)	0.079(A)	het	B(0.099)	D(0.000)	N(0.38)
TAF1	PARK12	Dystonia-Parkinsonism, X-linked	none found	–	–	–	–	–	–	–	–
FBX07	PARK15	PD 15, autosomal recessive	none found	–	–	–	–	–	–	–	–
VPS35	PARK17	PD 17	none found	–	–	–	–	–	–	–	–
DNAJC6	PARK19	PD 19, juvenile-onset	none found	–	–	–	–	–	–	–	–
SYNJ1	PARK20	PD 20, early-onset	rs71640263	c.4215_4216insAATACT	p.Val1405_Leu1406insAsnThr	0.43(A)	8.3e-06(AAGTACT)	Het	NA	NA	NA
			rs2833929	c.3635-7G>T	NA	0.29(A)	0.288(A)	het	NA	NA	NA
			rs2254562	c.1001A>G	p.Lys334Arg	0.30(C)	0.293(C)	het	PD(0.992)	T(0.117)	N(-1.54)
PRKRA	DYT16	Dystonia 16	rs3997876	c.*3C>T	NA	–	0.241(A)	het	NA	NA	NA
			rs77419724	c.677T>A	p.Ile226Asn	–	0.163(T)	het	PD(0.996)	D(0.013)	D(-4.72)
			rs75862065	c.380C>T	p.Pro127Leu	–	0.098(A)	het	PD(1.000)	D(0.01)	D(-8.60)
			rs61999302	c.173A>G	p.Asp58Gly	–	0.043(C)	het	B(0.367)	D(0.172)	D(-2.48)
			rs62176112	c.32C>T	p.Pro11Leu	0.0457 (A)	0.125 (A)	het	B (0.001)	T (0.134)	N (0.72)
			rs141354030	c.22_23delGC	p.Ala8Argfs	0.238 (G)	0.364 (GC)	het	NA	NA	NA
			rs9406386	c.1A>C	p.Met1Leu	–	0.462 (G)	het	B (0.000)	D (0.000)	N (-0.77)
			rs62176113	c.-24G>A	NA	–	0.276 (C)	het	NA	NA	NA
			rs80197731	c.-133C>G	NA	–	–	het	NA	NA	NA
			rs797021523	c.-146C>G	NA	–	–	het	NA	NA	NA
SLC6A3	–	Parkinsonism-dystonia, infantile	none found	–	–	–	–	–	–	–	–
SLC30A10	–	Hypermanganesemia with dystonia, polycythemia, and cirrhosis	none found	–	–	–	–	–	–	–	–
SLC39A8	–	Hypomanganesemia, CDG IIn	rs151371	c.*270G>A	NA	0.499 (T)	–	het	NA	NA	NA
			rs11382393	c.*262_263insC	NA	0.231 (G)	–	hom	NA	NA	NA
			rs35608353	c.*258G>A	NA	0.498 (T)	–	het	NA	NA	NA
			rs151370	c.*88T>C	NA	0.424 (A)	–	het	NA	NA	NA
**Patient E-II-4**											
SCNA	PARK1/ PARK4	PD 1/PD 4	none found	–	–	–	–	–	–	–	–
PARK2	PARK2	PD, juvenile, type 2	none found	–	–	–	–	–	–	–	–
PINK1	PARK6	PD 6, early onset	rs2298298	c.388-7A>G	NA	0.18 (A)	0.8623 (G)	hom	NA	NA	NA
			rs3131713	c.960-5G>A	NA	0.18 (G)	0.8628 (A)	hom	NA	NA	NA
PARK7	PARK7	PD 7, autosomal recessive early-onset	none found	–	–	–	–	–	–	–	–
LRRK2	PARK8	PD 8	rs2256408	c.149G>A	p.Arg50His	0.03 (G)	0.9911 (A)	hom	B (0.000)	T (1.000)	N (0.94)
			rs10878307	c.2167A>G	p.Ile723Val	0.04 (G)	0.05922 (G)	hom	B (0.001)	T (0.805)	N (0.11)
ATP13A2	PARK9	Kufor-Rakeb syndrome	novel	c.1180+7C>G*	NA	–	–	het	NA	NA	NA
TAF1	PARK12	Dystonia-Parkinsonism, X-linked	none found	–	–	–	–	–	–	–	–

FBX07	PARK15	PD 15, autosomal recessive	none found	–	–	–	–	–	–	–	–
VPS35	PARK17	PD 17	none found	–	–	–	–	–	–	–	–
DNAJC6	PARK19	PD 19, juvenile-onset	none found	–	–	–	–	–	–	–	–
SYNJ1	PARK20	PD 20, early-onset	rs844988	c.1201-3A>T	NA	0.11 (A)	0.08051 (A)	het	NA	NA	NA
PRKRA	DYT16	Dystonia 16	none found	–	–	–	–	–	–	–	–
SLC6A3	–	Parkinsonism-dystonia, infantile	none found	–	–	–	–	–	–	–	–
SLC30A10	–	Hypermanganesemia with dystonia, polycythemia, and cirrhosis	none found	–	–	–	–	–	–	–	–
SLC39A8	–	Hypomanganesemia, CDG IIn	none found	–	–	–	–	–	–	–	–

Supplementary Table 1. Variants in known early onset Parkinson's disease (PD) and Mn transporter disease genes identified on whole exome sequencing MAF: minor allele frequency; NA: not applicable; het: heterozygous; hom: homozygous; B: benign; N: neutral; T: tolerated; *Inappropriate segregation of this variant within this family confirmed by Sanger sequencing.

		White matter involvement[1]	Deep grey matter involvement (T1)				Deep grey matter involvement (T2)			Pituitary gland involvement	Cerebral/ cerebellar atrophy
		Generalized hyperintensity on T1	*Hyperintensity on T1*				*Hypointensity on T2*			*Hyperintensity on T1*	
			Caudate	Putamen	Globus pallidus	Thalamus	Globus pallidus				
		Axial/Sagittal images	Axial images				Axial images			Sagittal images	Sagittal/Coronal images
	**Age**						T2	T2*	Flair		
**A-II-1**	3	**++**[2]	**+**	**+**	**++**	**-**	**++**	N/A	**++**	**+/-**	**-**
	6	**++**[2]	**++**	**++**	**+++**	**-**	**+**	**++**	**+**	**+**	**+**
	9	**+++**[2]	N/A	N/A	N/A	N/A	**+**	**+++**	**+**	**+**	**++**
**B-II-4**	2	**++**	**+**	**+**	**++**	**-**	**+**[3]	N/A	**++**	**+**	**-**
**C-II-2**	3	**++**	**+**	**+**	**++**	**-**	**++**	N/A	**++**	**++**	**-**
**D-II-1**	3	**+++**			**+++**	**-**				**+**	**+++**
**E-II-2**	2	**++**	**+**	**+**	**++**	**-**	**++**	**+**	N/A	**++**	**-**
	4	**++**	**+**	**+**	**++**	**-**	**+**	**+/-**	N/A	**++**	**-**
	17	**++**	**++**	**++**	**++**	**-**	**++**	**+**	N/A	**++**	**-**
**E-II-3**	2	**++**	**+/-**	**+/-**	**++**	**-**	**+**	N/A	N/A	**+**	**-**

Supplementary Table 2. Neuroradiological features of individuals with mutations in *SLC39A14*

[1]Generalized white matter changes including involvement of the cerebellum, spinal cord, dorsal pons with sparing of the ventral pons

[2]Evidence of progressive cerebral atrophy especially involving frontal lobe and corpus callosum

[3]T2-weighted hypointensity associated with a central hyperintensity, similar to the eye-of-the-tiger sign, possibly due to "T1-shine through"

N/A – not available; (-) radiological feature absent; (+/-) radiological feature borderline; (+) radiological feature present; (++) radiological feature strongly present; (+++) radiological feature very strongly present

Primer	Sequence (5' → 3')	Amplicon size (bp)	Annealing temperature (°C)
**hSLC39A14_Exon2Fw**	TCAAGAAGGAGCAGAGAAGCA	480	60⁺/62*
**hSLC39A14_Exon2Rv**	AGACAGGGAACCCTGAGAGG		
**hSLC39A14_Exon3Fw**	TCCTCTGGGAAGGCTGAGTA	371	62⁺/60*
**hSLC39A14_Exon3Rv**	CATTCAGTGAGGAGCAGCAG		
**hSLC39A14_Exon4AFw**	GAGTGTCCCCACCCTCAGT	298	60⁺/62*
**hSLC39A14_Exon4ARv**	GTAGGGGAGGAGGGGATTG		
**hSLC39A14_Exon4BFw**	GGCATGTGCCTTCTCTCC	300	60
**hSLC39A14_Exon4BRv**	CCTTCTATCCAAACGGAGGTC		
**hSLC39A14_Exon5Fw**	AGGGGGATCAGTAAAGATGCT	242	60
**hSLC39A14_Exon5Rv**	TGTTTGAGATGGGTGTTTTCC		
**hSLC39A14_Exon6Fw**	AGCAGGTGCTCAATCAGGTT	328	60
**hSLC39A14_Exon6Rv**	ACCATGTGCCCTCAAGGTAA		
**hSLC39A14_Exon7Fw**	GGCTTACCTTGAGGGCACAT	377	60
**hSLC39A14_Exon7Rv**	GCACTGTGAAGACAGGGAGA		
**hSLC39A14_Exon8Fw**	CCATGCCCATCTTACTCTTCC	299	60
**hSLC39A14_Exon8Rv**	ACCTAACATCCATCCCCTTAG		
**hSLC39A14_Exon9AFw**	TTGCCCTGGACTTACAAGATG	297	60
**hSLC39A14_Exon9ARv**	GTGGTGCATTGTGGATGGT		
**hSLC39A14_Exon9BFw**	CGGCCATGTTTATGTTTTTG	299	60
**hSLC39A14_Exon9BRv**	CATCATGCAGTTAGGAAATACCA		

Supplementary Table 3. Primer sequences used for PCR and sequencing of human *SLC39A14*

Primers cover the transcripts NM_001128431.2, NM_015359.4 and NM_001135154.1. PCR was performed using either Taq DNA polymerase (Life Technologies) with the addition of Betaine (0.5M), cycling conditions: 95°C for 5 minutes; 35 cycles of 95°C for 30 seconds, specified annealing temperature ($^{+}$) for 30 seconds, 72°C for 30 seconds; final cycle 72°C for 5 minutes; or Biomix Red (Bioline), cycling conditions: 95°C for 5 minutes, 35 cycles of 95°C for 45 seconds, specified annealing temperature (*) for 45 seconds, 72°C for 1 minute; final cycle 72°C for 5 minutes.

Primer	Sequence (5' → 3')	Amplicon size (bp)	Annealing temperature (°C)
**hSLC39A14_RT_Exon3Fw**	CGGAGAACCAGGAAAACGAGG	109	64
**hSLC39A14_RT_Exon4aRv**	GAGGCCAGGTTAATCAGTGAG		
**hSLC39A14_RT_Exon4bFw**	ACAAGAGGCTGCTGCTCTAC	139	64
**hSLC39A14_RT_Exon5Rv**	CCAAACACCACTGCAGACTTGG		
**hHPRT_Fw**	CCACGAAAGTGTTGGATATAAGC	205	58
**hHPRT_Rv**	GGCGATGTCAATAGGACTCCAGA		
**zfSLC39A14_RT_Exon3Fw**	CCCTAGACCCACTGAAGCTG	240	58
**zfSLC39A14_RT_Exon5Rv**	GCGGACTTGGGCACATAATA		

Supplementary Table 4. Primer pairs for RT-PCR of human and zebrafish *SLC39A14*

Q5 High Fidelity DNA polymerase (NEB) was used with the addition of Q5 High GC Enhancer at 1X final concentration. Cycling conditions: 98°C for 30 seconds; 35 cycles of 98°C for 10 seconds, specific annealing temperature for 30 seconds, 72°C for 30 seconds per kb; final cycle 72°C for 2 minutes.

Primer	Sequence (5' → 3')	Amplicon size (bp)	Amplification efficiency (%)
**zfSLC39A14_isoform1_Fw**	CCTTTTACAAGCGGTTGCTGC	133	97.6
**zfSLC39A14_isoform1_Rv**	GCGGACTTGGGCACATAATA		
**zfSLC39A14_isoform2_Fw**	CGCGGTTCATGCGCAGAGTT	136	97.8
**zfSLC39A14_isoform2_Rv**	GCGGACTTGGGCACATAATA		
**zfSLC39A14_all_isoforms_Fw**	CCCTAGACCCACTGAAGCTG	240	101.5
**zfSLC39A14_all_isoforms_Rv**	GCGGACTTGGGCACATAATA		
**zfEf1alpha_Fw**	GTACTTCTCAGGCTGACTGTG	136	98.2
**zfEf1alpha_Rv**	ACGATCAGCTGTTTCACTCC		
**zfDMT1_Ex1_2_Fw**	TTCCCAGCAAACAACGAGAC	101	106.9
**zfDMT1_Ex1_2_Rv**	CCCACAGCCCAGATGTAGAG		
**zfSlc39a8_Ex2_3_Fw**	GCCTGCCCTTACACTTCTTC	104	109.4
**zfSlc39a8_Ex2_3_Rv**	GCAGCCAAGTTAATCACCGT		
**Dr_tfr1a_1_SG QuantiTect Primer Assay (Qiagen, QT02189621)**			
**Dr_tfr1b_1_SG QuantiTect Primer Assay (Qiagen, QT02196558)**			

Supplementary Table 5. Primers used for real-time PCR

Primer	Sequence (5’ → 3’)
**InfFw_pCS2_hSLC39A14**	GCCTCTCGAGCCTCTAGAACT
**InfRv_pCS2_hSLC39A14**	CCCAATCTGGATCTGTCCTGA
**InfFW_EGFP**	CAGATCCAGATTGGGGGTGGATCAGGAGGTGGC
**InfRV_EGFP**	AGAGGCTCGAGAGGCTTACTTGTACAGCTCGTCCATGC

Supplementary Table 6. Primers used to generate EGFP tagged constructs of human *SLC39A14* at the C-terminus. 15 bp overhangs complementary to the ends of the linearized vector are underlined.

Mutation	Sequence (5’ → 3’)
**c.292T>G Fwd**	TTAGTTCTGGAGACCTCGTCACTGCCCACA
**c.292T>G Rev**	GAAATTGTGGGCAGTGACGAGGTCTCCAGA
**c.1147G>A Fwd**	GTTCCCACATGAGCTAAGAGACTTTGTCATCC
**c.1147G>A Rev**	GGATGACAAAGTCTCTTAGCTCATGTGGGAAC
**c.1407C>G Fwd**	TGATTCCATTTATCATCCAGAAGCTGGGCCTCCTG
**c.1407C>G Rev**	CAGGAGGCCCAGCTTCTGGATGATAAATGGAATCA

Supplementary Table 7. Primers used for site-directed mutagenesis. Altered nucleotide is underlined.

	Sequence (5’ → 3’)
**zfSLC39A14_Fw**	TCTCTGTGTTCACTGGTCGG
**zfSLC39A14_Rv_T7**	TAATACGACTCACTATAGGGTGGTGAGTACAAGCATTATGGC

Supplementary Table 8. Primers used to generate an antisense DIG labelled RNA probe for whole mount *in situ* hybridisation. Q5 High Fidelity DNA polymerase (NEB) was used for PCR amplification (annealing temperature 58°C) according to the manufacturer’s recommendations. T7 promoter sequence underlined.

	Sequence (5’ → 3’)
**5’ RACE zfSLC39A14 outer**	CCAGCGCAATGTAGAGGAAC
**5’ RACE zfSLC39A14 inner**	GCAGGATCACAAAATCACCCA
**3’ RACE zfSLC39A14 outer**	ACTAATCCCAGAGGCCTTCG
**3’ RACE zfSLC39A14 inner**	CATGGGCACAGTCACTTTCC

Supplementary Table 9. Zebrafish *slc39a14* specific primers used for 5’ and 3’ RACE

Supplementary Note 1

Case descriptions

Family A

Family A are of Yemeni origin. Parents are distantly related with two affected daughters. Prenatal course, delivery and early infancy were normal for both siblings. The older sibling was reported to have onset of severe neurological deterioration from seven months of age. Her prominent clinical features at the age of nine years included progressive spasticity, dystonia, intellectual disability and microcephaly. She was using a wheelchair, made eye contact and tracked with her eyes, and had dystonia particularly affecting her mouth, face and neck. She had increased tone in all four limbs with prominent spasticity, especially at the ankles. Reflexes were brisk throughout with evidence of bilateral ankle clonus. A conjunctival biopsy at 18 months was normal. The younger female sibling had disease onset in infancy at a similar age to her older sister (six months). Examination at seven years old revealed that she was alert and responsive, seated in a supportive wheelchair, non-ambulatory, non-verbal, and unable to follow commands. She tracked with her eyes. She did not have the facial dystonia described in her older sister. Her facial movements were scant but symmetrical. She also had significant spasticity in all four limbs and increased reflexes and clonus in both ankles. She died at the age of eight years from a respiratory infection.

Family B

Family B originate from Egypt. The parents are first cousins and have two affected and two unaffected daughters. Both affected siblings were born by normal vaginal delivery and had an uneventful peri- and postnatal period. Following a normal initial neurodevelopment, both siblings presented with loss of previously acquired motor developmental milestones associated with increased irritability at seven months of age. This was followed by the development of generalized dystonia and bulbar dysfunction, bradykinesia, and axial hypotonia. No pyramidal tract signs were noted, and hearing and vision appeared normal. The older sibling passed away at the age of thirteen months. The cause of death is unknown. Unfortunately, the family was lost to follow up after the initial consultation and we have no further information on the progress of the second affected sibling.

Family C (Supplementary Movie 1-2)

Parents are second degree cousins from India and have one affected and one unaffected child. The affected girl was born by elective caesarean section and had an uneventful postnatal period. Her early developmental milestones were normal. At the age of three years she presented with clumsiness while writing and drawing. Shortly after, she developed toe walking and lost her ability to stand and walk unaided. By five years of age, she showed marked hypomimia, dystonic posturing of both lower limbs, tremor in her upper limbs and dyskinetic movements in both hands. She had increased tone in all four limbs associated with hyperreflexia, ankle clonus, bilateral extensor plantar reflexes and bilateral tendoachilles contractures. There were no cerebellar signs.

Intellectual ability was normal. There was no history of environmental Mn exposure. Nerve conduction studies and visual evoked potentials were normal. Treatment with baclofen, tetrabenazine and levodopa did not lead to significant improvement of symptoms. Once hypermanganesemia was confirmed she was commenced on monthly $Na_2CaEDTA$ infusions. Chelation treatment lead to cessation of tremors and dyskinetic movements in her upper limbs and improvement of lower limb dystonic posturing. Despite residual dystonia in her lower limbs she regained the ability to walk independently wearing ankle foot orthoses. Currently, she receives monthly five day courses of $Na_2CaEDTA$ (500 mg twice daily), Zincovit (5 mL twice daily), Evion (400 mg daily), Vitamin C (500 mg daily), Tetrabenazine (6.25/12.5 mg twice daily), Baclofen (5 mg twice daily) and Tonoferon (5 mL daily).

Family D

The affected boy who is of Spanish ancestry is the only child of first cousin parents. He was born by normal vaginal delivery following an uncomplicated pregnancy. He presented with motor developmental delay, axial hypotonia, hypokinesia and macrocephaly at the age of ten months. This was followed by the development of generalized dystonia, lower limb hypertonia, irritability and episodic hyperthermia with hyperhidrosis. He suffered from dystonic crises with opisthotonus. He showed minimal improvement on levodopa, trihexyphenidyl, gabapentin and clonazepam. He did not achieve language development. He died at four years of age from *E. coli* septic shock with multiorgan failure following bronchopneumonia. Hypermanganesemia was persistent with values four to twenty times that of normal. Neither parent had hypermanganesemia and environmental Mn exposure was ruled out. He was found to have elevated creatine kinase (from normal to 3,023 U L^{-1}) on occasions with normal electromyography (EMG). Metabolic screening included plasma amino acids, urine organic acids, CSF neurotransmitter amine metabolites and transferrin isoelectric focusing, all of which was negative. The only metabolic abnormality found was an impairment of the mitochondrial respiratory chain with deficiencies of complex III and V in muscle. Molecular analysis for recessive mitochondrial DNA mutations was negative.

Family E (Supplementary Movie 3-5)

Family E are of Lebanese origin with consanguineous first cousin parents. There are three affected children (17 year old girl, 16 year old girl and a 9 year old boy) and three unaffected children (all boys). All children were born following a normal pregnancy and birth and developmental milestones were normal until two to three years of age. All three affected children show a homogeneous clinical presentation predominated by a progressive and severe generalized dystonia. Initial presentation in all three children was with an abnormality in gait (unsteadiness and difficulty in independent walking) associated with lower limb dystonia, with features of symmetrical lower limb postural dystonia, toe walking, scissoring and plantar flexion. Over the next six to twelve months ambulation became increasingly difficult and the dystonia became more generalized in nature with involvement of the upper limbs (dystonic posturing, fisting). Oromandibular dystonia was also a prominent feature in all three children. Loss of ambulation occurred between seven and ten years of age and all children

developed limb contractures and scoliosis. All three children developed acquired microcephaly. There were no cerebellar features clinically and neuropsychiatric features have not been evident. There appears to be relative cognitive sparing (psychometric testing has not been possible) although a degree of learning disability is present in all children. Ophthalmological assessment, VER and ERG were unremarkable. Nerve conduction and EMG were also normal. None of the children have shown clinical response to medication including levodopa up to 5 mg kg^{-1} per day and benzodiazepines. There has been partial but poorly sustained response to trihexyphenidyl at high doses of 20 mg per day and intrathecal baclofen of 1,500-2,000 µg per day in the two older siblings.
